# Bioactive Ingredients in Chinese Herbal Medicines That Target Non-coding RNAs: Promising New Choices for Disease Treatment

**DOI:** 10.3389/fphar.2019.00515

**Published:** 2019-05-21

**Authors:** Yan Dong, Hengwen Chen, Jialiang Gao, Yongmei Liu, Jun Li, Jie Wang

**Affiliations:** Department of Cardiology, Guang'anmen Hospital, China Academy of Chinese Medical Sciences, Beijing, China

**Keywords:** bioactive ingredient, Chinese herbal medicine, traditional Chinese medicine, ncRNA, therapeutic target

## Abstract

Chinese herbal medicines (CHMs) are widely used in China and have long been a powerful method to treat diseases in Chinese people. Bioactive ingredients are the main components extracted from herbs that have therapeutic properties. Since artemisinin was discovered to inhibit malaria by Nobel laureate Youyou Tu, extracts from natural plants, particularly bioactive ingredients, have aroused increasing attention among medical researchers. The bioactive ingredients of some CHMs have been found to target various non-coding RNA molecules (ncRNAs), especially miRNAs, lncRNAs, and circRNAs, which have emerged as new treatment targets in numerous diseases. Here we review the evidence that, by regulating the expression of ncRNAs, these ingredients exert protective effects, including pro-apoptosis, anti-proliferation and anti-migration, anti-inflammation, anti-atherosclerosis, anti-infection, anti-senescence, and suppression of structural remodeling. Consequently, they have potential as treatment agents in diseases such as cancer, cardiovascular disease, nervous system disease, inflammatory bowel disease, asthma, infectious diseases, and senescence-related diseases. Although research has been relatively limited and inadequate to date, the promising choices and new alternatives offered by bioactive ingredients for the treatment of the above diseases warrant serious investigation.

## Introduction

Chinese herbal medicines (CHMs) were the main treatment method used in ancient times by the Chinese to combat disease. As early as the Qin and Han Dynasty (around 221 BCE to 220 CE), *Sheng Nong's Herbal Classic* recorded 365 medicines. By the time of the Ming Dynasty (1368–1644), the number of CHMs listed in the book of *Compendium of Materia Medica* had increased to 1892. Most herbal medicines in such publications have been used constantly throughout medical history and are still applied in practice today. For example, according to *Sheng Nong's Herbal Classic, Coptis chinensis* Franch. was found to relieve abdominal pain and diarrhea and this herb is still widely used in China for the treatment of diarrhea or dysentery. Further, *Panax notoginseng* (Burk.) F. H. Chen, a traditional herb was initially used to stop bleeding, promote blood circulation and ease pain, was recorded in the *Compendium of Materia Medica*. It is now commonly used in cases of trauma and cardiovascular, and cerebrovascular diseases. A recent meta-analysis further demonstrated that several *P. notoginseng* preparations are beneficial for patients with unstable angina pectoris (Song et al., 2017).

Despite the positive effects of CHMs, little is known about their effective constituents, bioactive ingredients, and mechanisms of action. Therefore, in addition to the clinical applications mentioned in classic texts, understanding the specific active ingredients and clarifying the mechanisms of action of these compounds would facilitate the improved application of CHMs. The discovery of the drug artemisinin best illustrates the importance of CHM to the world (Tu, 2016), inspiring the notion that, through study of their bioactive ingredients, CHMs can help people around the world to conquer life-threatening diseases.

Non-coding RNA molecules (ncRNAs), which mainly comprise miRNA, lncRNA, and circRNA, do not encode proteins; however, as the most abundant class of RNA (at least 90%) (Sana et al., 2012), ncRNAs have important functions in gene regulation and are involved in pathological processes contributing to many diseases (Batista and Chang, 2013; Memczak et al., 2013; Zhang et al., 2018a), particularly cancer, and cardiovascular and nervous system diseases. Moreover, circRNA and lncRNA act as competitive endogenous RNAs (ceRNA), which are natural miRNA sponges that influence miRNA-induced gene silencing via miRNA response elements (Tay et al., 2014). Thus, complex regulatory networks exist, comprising circRNA, lncRNA, miRNA, and target genes. Unraveling of this complexity has laid the foundation for a comprehensive understanding of the pathology and treatment of diseases influenced by gene regulatory networks, rather than only core disease-related genes (Boyle et al., 2017). Excitingly, recent studies (Feng et al., 2015; Tian F. et al., 2017; Zhou Y. et al., 2017) have revealed that some miRNA, lncRNA, circRNA, and ceRNA crosstalk can be regulated by bioactive ingredients from CHMs, which often have multiple targets (Table 1). By influencing regulatory mechanisms, including pro-apoptosis (Feng et al., 2015), anti-proliferation and anti-migration (Liu T. et al., 2017), anti-inflammation (Fan et al., 2016), anti-atherosclerosis (Han et al., 2018), anti-infection (Liu et al., 2016), anti-senescence (Zhang J. et al., 2017), and suppression of structural remodeling (Liu L. et al., 2017), these ingredients exert protective functions in cancer, cardiovascular disease, nervous system disease, inflammatory bowel disease, asthma, infectious diseases, and senescence-related diseases.

**Table 1:** Detailed information on bioactive ingredients targeting ncRNAs.

**No**.	**Name of ingredient**	**Original plant**	**ncRNA target**	**Gene/protein/pathway**	**Disease**
1	Berberine	*Coptis chinensis* Franch., *Berberis soulieana* Schneid, *Berberis poiretii* Schneid., *Berberis vernae* Schneid, *Berberis wilsoniae* Hemsl., and *Platycladus orientalis* (Linn.) Franco.	miR-99a~125b (Feng et al., 2015) miR-21 (Luo et al., 2014) miR-19a/92a (Yin et al., 2018)	RAC1, NFκB1, MYC, JUN, and CCND1 (Feng et al., 2015) IL6/STAT3, PDCD4 (Luo et al., 2014) P53 signaling pathway (Luo et al., 2014) P53, ERB, and MAPK signaling pathways (Feng et al., 2015)	Multiple myeloma (Luo et al., 2014; Feng et al., 2015; Yin et al., 2018)
			miR-23a (Wang N. et al., 2014)	NEK6, P53, P21, GADD45α (Wang N. et al., 2014)	Hepatocellular carcinoma (Wang N. et al., 2014)
			miR-152 miR-429 miR-29a (Huang et al., 2017) miR-296-5p (Su et al., 2015)	DNMT1, DNMT3A, DNMT3B, CDK4 (Su et al., 2015; Huang et al., 2017) Pin1-β-catenin-cyclin D1 signaling pathway (Su et al., 2015)	Colorectal cancer (Su et al., 2015; Huang et al., 2017)
			miR-34a miR-154 miR-26a miR-124 (Yang L. H. et al., 2016)	CDK4, CyclinD1, CyclinE, CDK2 (Yang L. H. et al., 2016)	Melanoma (Yang L. H. et al., 2016)
			miR-93 (Chen et al., 2015)	miR-93/PTEN/AKT signaling pathway (Chen et al., 2015)	Ovarian cancer (Chen et al., 2015)
			miR-203 (You et al., 2016)	BCL-w (You et al., 2016)	Gastric cancer (You et al., 2016)
			miR-27a miR-27b (Wu et al., 2016)	PPAR-γ (Wu et al., 2016)	Obesity (Wu et al., 2016)
			lncRNA MRAK052686 (Yuan et al., 2015)	NRF2 (Yuan et al., 2015)	Steatotic liver (Yuan et al., 2015; Li C. H. et al., 2018)
			miR-373 (Li C. H. et al., 2018)	EGR1, AKT1 AKT-mTOR-S6K signaling pathway (Li C. H. et al., 2018)	
			miR-29a-3p (Mao et al., 2018)	IRS1 (Mao et al., 2018)	Insulin resistance (Mao et al., 2018)
2	Artesunate	*Artemisia annua* L.	lncRNA UCA1 miR-184 (Zhou S. et al., 2017)	lncRNA UCA1/miR-184/BCL-2 axis (Zhou S. et al., 2017)	Prostate cancer (Zhou S. et al., 2017)
3	Triptolide/ Triptonide	*Tripterygium wilfordii* Hook. f	miR-21 (Li et al., 2016)	Caspase-3 and 9, PTEN (Li et al., 2016)	Non-small cell lung cancer (Li et al., 2016)
			227 miRNAs (Reno et al., 2015)	Focal adhesion kinase (Reno et al., 2015)	Lung cancer (Reno et al., 2015)
			miR-17-92 miR-106b-25 (Li S. G. et al., 2018)	c-MYC, BIM, PTEN, and P21 (Li S. G. et al., 2018)	Hepatocellular carcinoma (Li S. G. et al., 2018)
			lncRNA THOR (Wang et al., 2019)	IGF2BP1, Myc, IGF2, and Gli1 (Wang et al., 2019)	Nasopharyngeal carcinoma (Wang et al., 2019)
4	Ailanthone	*Ailanthus altissima* (Mill.) Swingle	miR-21 (Yang P. et al., 2018)	caspase 3, caspase 9, Beclin-1, LC3-II, p62, and cyclin D1 Ras/Raf/MEK/ERK and mTOR pathways (Yang P. et al., 2018)	Vestibular schwannomas (Yang P. et al., 2018)
5	Cordycepin/Soya-cerebroside	*Cordyceps militaris*	miR-21 (Yang et al., 2017)	PTEN, Akt (Yang et al., 2017)	Renal cell carcinoma (Yang et al., 2017)
			miR-432 (Liu S. C. et al., 2017)	MCP-1 AMPK and AKT signaling pathways (Liu S. C. et al., 2017)	Osteoarthritis (Liu S. C. et al., 2017)
6	Tubeimoside I	*Bolbostemma paniculatum* (Maxim) Franquet	miR-126-5p (Shi et al., 2017)	VEGF-A/VEGFR-2/ERK signaling pathway (Shi et al., 2017)	Non-small cell lung cancer (Shi et al., 2017)
7	Oridonin	*Rabdosia rubescens* (Hemsl.) Hara	105 miRNAs (Gui et al., 2015)	/	Laryngeal cancer (Gui et al., 2015)
8	Curcumin	*Curcuma longa* L.	miR-208 (Guo H. et al., 2015)	CDKN1A (Guo H. et al., 2015)	Prostate cancer (Guo H. et al., 2015; Cao et al., 2017; Liu W. L. et al., 2017; Zhang et al., 2018b)
			miR-145 lncRNA-ROR (Liu W. L. et al., 2017)	CCND1, CDK4, OCT4, CD44, and CD133 (Liu W. L. et al., 2017)	
			miR-770-5p miR-1247 (Zhang et al., 2018b)	DLK1-DIO3 (Zhang et al., 2018b)	
			miR-143 (Cao et al., 2017)	PGK1, FOXD3 (Cao et al., 2017)	
			miR-98 (Liu W. L. et al., 2017)	LIN28A, MMP 2, MMP9 (Liu W. L. et al., 2017)	Lung cancer (Tang et al., 2010; Liu W. L. et al., 2017)
			miR-186* (Tang et al., 2010)	/	
			miR-203 (Saini et al., 2011)	AKT2, SRC (Saini et al., 2011)	Bladder cancer (Saini et al., 2011)
			miR-33b (Sun et al., 2016)	XIAP (Sun et al., 2016)	Gastric cancer (Sun et al., 2016)
			miR-192-5p (Jin et al., 2015)	PI3K/AKT signaling pathway (Jin et al., 2015)	Non-small cell lung cancer (Jin et al., 2015)
			miR-7 (Ma et al., 2014)	SET8 (Ma et al., 2014)	Pancreatic cancer (Ma et al., 2014)
			miR-30c (Lu et al., 2017)	MTA1 (Lu et al., 2017)	Paclitaxel-resistant non-small-cell lung cancer (Lu et al., 2017)
			miR-29b-1-5p (Zhou S. et al., 2017)	PPARG, RRM2, SRSF1, EPAS1, MAPK, mTOR, PI3K-AKT, AMPK, TNF RAS signaling pathways (Zhou S. et al., 2017)	Adriamycin-resistant breast cancer (Zhou S. et al., 2017)
			lncRNA AK294004 (Wang Q. et al., 2014)	Cyclin D1(Wang Q. et al., 2014)	Radioresistant nasopharyngeal carcinoma (Wang N. et al., 2014)
			miR-146a (Wu et al., 2015) miR-378 (Li et al., 2017)	NF-κB signaling (Wu et al., 2015)P38 (Li et al., 2017)	Glioblastoma (Wu et al., 2015; Li et al., 2017)
			miR-34a (Guo et al., 2013)	BCL-2, BMI-1 (Guo et al., 2013)	Breast cancer (Guo et al., 2013; Zhou et al., 2018)
			miR-29b-1-5p miR-29b-3p miR-6068 miR-6790-5p miR-4417 (Zhou et al., 2018)	DDIT4, EPAS1, VEGFA, RPS14, and DCDC2 (Zhou et al., 2018)	
			miR-122 miR-221 (Zhang S. et al., 2017)	FGF2, MMP2, VEGF, HGF, TF, FVII (Zhang S. et al., 2017)	Hepatocellular Carcinoma (Guo Y. et al., 2015; Liu W. L. et al., 2017)
			lncRNA AK125910 (Guo Y. et al., 2015)		
			miR-155 (Ma F. et al., 2017)	TNF-α, IL-6 PI3K/AKT pathway (Ma F. et al., 2017)	LPS-induced inflammatory response (Ma F. et al., 2017)
			miR-17-5p (Tian L. et al., 2017)	WNT signaling pathway effector TCF7l2 (Tian L. et al., 2017)	Adipogenic differentiation (Tian L. et al., 2017)
9	Shikonin	*Lithospermum erythrorhizon* Sieb. et Zucc.	miR-106b (Huang and Hu, 2018)	miR-106b/PTEN/AKT/mTOR signaling pathway (Huang and Hu, 2018)	Endometrioid endometrial cancer (Huang and Hu, 2018)
			miR-128 (Wei et al., 2016)	BAX (Wei et al., 2016)	Breast cancer (Wei et al., 2016)
			miR-143 (Liu et al., 2015)	BAG3 (Liu et al., 2015)	Glioblastoma (Liu et al., 2015)
10	Paeoniflorin	*Paeonia lactiflora* Pall.	miR-16 (Li W. et al., 2015)	MMP-9 (Li W. et al., 2015)	Glioma (Li W. et al., 2015)
11	Honokiol	*Magnolia grandiflora*	miR-34a (Avtanski et al., 2015)	STAT3 WNT1-MTA1-β-catenin signaling (Avtanski et al., 2015)	Breast tumor (Avtanski et al., 2015)
12	Schisandrin B	*Schisandra sphenanthera* Rehd. et Wils.	miR-150 lncRNA BCYRN1 (Zhang X. Y. et al., 2017)	miR-150/ lncRNA BCYRN1/ cell proliferation axis (Zhang X. Y. et al., 2017)	Asthma (Lu et al., 2016)
13	Resveratrol	Grapes, blueberries, *Morus alba* L., *Polygonum cuspidatum* Sieb. et Zucc. and *Rubus idaeus* L.	lncRNA MALAT1 (Ji et al., 2013)	c-MYC, MMP-7 WNT/β-catenin signaling (Ji et al., 2013)	Colorectal cancer (Ji et al., 2013; Karimi Dermani et al., 2017)
			miR-200c (Karimi Dermani et al., 2017)	Vimentin, ZEB-1, E-cadherin (Karimi Dermani et al., 2017)	
			miR-221 (Wu and Cui, 2017)	NF-κB, TFG (Wu and Cui, 2017)	Melanoma (Wu and Cui, 2017)
			miR-21 miR-30a-5p miR-19 (Wang G. et al., 2015)	P53, PTEN, EGFR, STAT3, COX-2, NF-κB PI3K/AKT/mTOR pathway (Wang G. et al., 2015)	Glioma (Wang G. et al., 2015)
			miR-155 (Ma C. et al., 2017)	TNF-α, IL-6, MAPKs, STAT1/STAT3, and SOCS1 (Ma C. et al., 2017)	LPS-induced inflammatory response (Ma C. et al., 2017)
			miR-663 miR-155 (Tili et al., 2010)	JUNB, JUND, activator protein-1 (Tili et al., 2010)	Malignancies (Tili et al., 2010)
			miR-96 (Bian et al., 2017)	BAX (Bian et al., 2017)	Hypoxia/ischemia-induced brain injury (Bian et al., 2017)
			miR-13 miR-124 (Zhao et al., 2013)	CREB, BDNF (Zhao et al., 2013)	Alzheimer's disease (Zhao et al., 2013)
14	Soybean Isoflavones	*Glycine max* (Linn.) Merr.	miR-29a miR-1256 (Li et al., 2012)	TRIM68, PGK-1 (Li et al., 2012)	Prostate cancer (Li et al., 2012)
15	Matrine	*Sophora flavescens Ait*	miR-19b-3p (Wei et al., 2018)	PTEN (Wei et al., 2018)	Melanoma (Wei et al., 2018)
16	Corylin	*Psoralea corylifolia* Linn	lncRNA GAS5 (Chen et al., 2018)	/	Hepatocellular carcinoma (Chen et al., 2018)
17	Tanshinone IIA/Magnesium lithospermate B	*Salvia miltiorrhiza* Bge.	miR-155 miR-147 miR-184 miR-29b miR-34c (Fan et al., 2016)	TLR4, MyD88, GM-CSF, sICAM-1, CXCL-1, MIP-1α, TNF-α, IL-1β, COX-2 TLR4-NF-κB pathway (Fan et al., 2016)	LPS-induced inflammation (Fan et al., 2016)
			miR-146b miR-155 (Xuan et al., 2017)	CRP, ox-LDL, IL-1β, IL-6, IL-12, TNF-α, CCL-2, CD40, and MMP-2 (Xuan et al., 2017)	Atherosclerosis (Xuan et al., 2017)
			miR-133 (Zhang et al., 2012)	MAPK ERK1/2 (Zhang et al., 2012)	Hypoxia (Zhang et al., 2012)
			miR-1 (Shan et al., 2009; Zhang et al., 2010)	SRF, Kir2.1 (Shan et al., 2009)	Arrhythmias post-AMI (Shan et al., 2009)
				Cx43, SRF, MEF2, P38 MAPK signal pathway (Zhang et al., 2010)	Myocardial infarction (Zhang et al., 2010)
			miR-107 (Yang et al., 2015)	GLT-1, glutamate (Yang et al., 2015)	Cerebral I/R injury (Yang et al., 2015)
18	Baicalin	*Scutellaria baicalensis* Georgi	miR-191a (Wang L. et al., 2017)	ZO-1 (Wang L. et al., 2017)	Inflammatory bowel disease (Wang L. et al., 2017)
			miR-294 (Wang J. et al., 2017)	c-jun and c-fos (Wang J. et al., 2017)	Inhibition of proliferation (Wang J. et al., 2015)
19	Cinnamaldehyde	*Cinnamomum cassia* Presl.	miR-21 miR-155 (Qu et al., 2018)	TNF-α, IL-1β, IL-6, AKT, mTOR, COX2 (Qu et al., 2018)	Ulcerative colitis (Qu et al., 2018)
			has-circ-0043256, miR-1252 (Tian F. et al., 2017)	has-circ-0043256/miR-1252/ITCH axis WNT/β-catenin pathway (Tian F. et al., 2017)	Non-small cell lung cancer (Tian F. et al., 2017)
20	Geniposide	*Gardenia jasminoides Ellis*	miR-145 (Su et al., 2018)	IL-6, TNF-α, MCP-1, MEK/ERK pathway (Su et al., 2018)	Inflammation in cardiomyocyte (Su et al., 2018)
21	Carvacrol/ Thymol	*Origanum vulgare* L. or wild bergamot	miR-155 miR-146a miR-21 (Khosravi and Erle, 2016)	TLR2, TLR4, SOCS1, SHIP1 (Khosravi and Erle, 2016)	Asthma (Khosravi and Erle, 2016)
22	3-acetyl-11-keto-β-boswellic acid	*Boswellia serrata*	miR-155 (Sayed et al., 2018)	P-IκB-α, carbonyl protein, SOCS-1 (Sayed et al., 2018)	Neuroinflammation (Sayed et al., 2018)
23	Sinapic acid	*Sinapis alba* L.	LncRNA MALAT1 (Han et al., 2018)	ET-1, IL-1β, ASC, NRLP3, Caspase-1 (Han et al., 2018)	Diabetic atherosclerosis (Han et al., 2018)
24	Polydatin	*Polygonum cuspidatum* Sieb. et Zucc.	miR-214 (Zhou et al., 2016)	Blood glucose, ALT, AST, TC, TG, LDL-C, HDL-C, MDA, SOD (Zhou et al., 2016)	Atherosclerosis with liver dysfunction (Zhou et al., 2016)
25	Ampelopsin	*Ampelopsis grossedentata*	miR-21 (Yang D. et al., 2018)	eNOS, DDAH1, NO, and ADMA (Yang D. et al., 2018)	Endothelial dysfunction (Yang D. et al., 2018)
			miR-34a (Kou et al., 2016)	SIRT1-mTOR signal pathways (Kou et al., 2016)	Neurodegenerative diseases (Kou et al., 2016)
26	Icariine	*Epimedium brevicornu* Maxim.	miR-34c (Liu et al., 2016)	RUNX2, JNKs, and p38, NF-kB pathways (Liu et al., 2016)	Bacteria-induced bone loss diseases (Liu et al., 2016)
			miR-21 (Li J. et al., 2015)	PTEN, RECK, Caspase-3, and BCL-2 (Li J. et al., 2015)	Ovarian cancer (Li J. et al., 2015)
27	Ginsenosides	*Panax ginseng* C. A. Mey.	miR-15b (Chan et al., 2011)	IP-10 (Chan et al., 2011)	H9N2/G1 infection (Chan et al., 2011)
28	Salidroside	*Rhodiola rosea* L	let-7c let-7e miR-3620 miR-411 miR-24-2-5p miR-485-3p (Zhang J. et al., 2017)	p53, transcription factor CREB AKT/mTOR signaling (Zhang J. et al., 2017)	Senescence (Zhang J. et al., 2017)
29	Phlorizin	*Acanthopanax senticosus* (Rupr. et Maxim.) Harms	miR-135b (Choi et al., 2016)	p63, PCNA, integrin α6, integrin β1, and type IV collagen (Choi et al., 2016)	Skin aging (Choi et al., 2016)
30	Osthole	*Cnidium monnieri* (L.) Cuss.	miR-107 (Xiao et al., 2017)	Aβ, BACE1, and LDH (Xiao et al., 2017)	Alzheimer's disease (Xiao et al., 2017)
31	Panax Notoginseng Saponins	*Panax notoginseng* (Burk.) F. H. Chen.	miR-29c (Liu L. et al., 2017)	Collagen (Col) 1a1, Col1a2, Col3a1, Col5a1, FBN1, TGFβ1 (Liu L. et al., 2017)	Myocardial injury and fibrosis (Liu L. et al., 2017)
			miR-146b-5p (Wang J. et al., 2017)	/	Oxidative damage (Wang J. et al., 2017)
			miR-34a (Lai et al., 2018)	miR-34a/SIRT1/p53 pathway (Lai et al., 2018)	Senescence (Lai et al., 2018)
			miR-18a (Yang Q. et al., 2014)	CD34, VWF (Yang Q. et al., 2014)	Tumor complicated with myocardial ischemia (Yang Q. et al., 2014)
			miR-222 (Yang Q. et al., 2016)	p27 and PTEN Met/miR-222 axis (Yang Q. et al., 2016)	Lewis lung carcinoma (Yang Q. et al., 2016)
32	Tetrandrine	*Stephania tetrandra* S. Moore.	miR-27b miR-125b (Ning et al., 2016)	VEGFC, BCL2L12, COL4A3, FGFR2 (Ning et al., 2016)	Hypertrophic scar (Ning et al., 2016)
33	Leonurine	*Leonurus artemisia* (Laur.) S. Y. Hu F.	miR-1 (Lu et al., 2019)	ANP, ET-1, p38 MAPK, p-p38 MAPK, myocyte enhancer factor 2, β-myosin heavy chain, and α-myosin heavy chain protein (Lu et al., 2019)	Cardiomyocyte hypertrophy (Lu et al., 2019)
34	Calycosin/ Astragaloside IV/Total flavonoids	*Astragalus membranaceus* (Fisch.) Bunge.	miR-375 (Wang Y. et al., 2014)	ER-α and Bcl-2 and RASD1 (Wang Y. et al., 2014)	Cerebral I/R injury (Wang Y. et al., 2014)
			lncRNA EWSAT1 (Kong et al., 2018)	/	Nasopharyngeal carcinoma (Kong et al., 2018)
			miR-34a (Zhang C. et al., 2018)	LDHA, MCT1, MCT4, HIF-1α, CD147, TIGAR and p53 (Zhang C. et al., 2018)	Gastric carcinoma (Zhang C. et al., 2018)
			miR-378 miR-378* (Wan et al., 2017)	/	Viral myocarditis (Wan et al., 2017)
35	Paeonol	*Paeonia suffruticosa* Andr. and *Cynanchum paniculatum* (Bunge) Kitagawa	miR-1 (Zhang and Xiong, 2015)	/	Ischemic arrhythmia (Zhang and Xiong, 2015)
36	Salvianolic acid A	*Salvia miltiorrhiza* Bge.	miR-101 (Yu D. S. et al., 2017)	tight junction proteins, HO-1, p-caveolin-1, ZO-1, occluding, Nrf2, and Cul3 (Yu D. S. et al., 2017)	Spinal cord injury (Yu D. S. et al., 2017)
			miR-3686 miR-4708-3p miR-3667-5p miR-4738-3p (Chen et al., 2016)	MDR1 (Chen et al., 2016)	Lung cancer (Chen et al., 2016)
37	Andrographolide	*Andrographis paniculata* (Burm. f.) Nees	miR-222-3p miR-106b-5p miR-30b-5p miR-23a-5p (Lu et al., 2016)	signaling pathways of miRNAs in cancer, MPAKs, and focal adhesion (Lu et al., 2016)	Hepatoma tumor (Lu et al., 2016)
38	Puerarin	*Radix Puerariae Lobatae*	miR-22 (Wang L. et al., 2014)	caveolin-3, amphiphysin-2, and junctophinlin-2 (Wang L. et al., 2014)	Cardiovascular diseases (Wang L. et al., 2014)

## Methodology

The bioactive ingredients of CHMs and their interactions with ncRNA targets are the subject of intensive and rapidly expanding research. In this study, we undertook a comprehensive review of this research. The PubMed database was searched using the terms: “(ncRNA) AND herbal medicine”, “(((miRNA) OR lncRNA) OR circRNA) AND herbal medicine,” “(((miRNA) OR lncRNA) OR circRNA) AND active ingredient,” “(((miRNA) OR lncRNA) OR circRNA) AND Chinese herb,” “(((miRNA) OR lncRNA) OR circRNA) AND natural agent,” “(((miRNA) OR lncRNA) OR circRNA) AND natural compound,” or “(((miRNA) OR lncRNA) OR circRNA) AND traditional Chinese medicine.”

In addition, the China National Knowledge Infrastructure (CNKI) was also searched with terms as follows: “FT = ‘Chinese herbal medicine’ AND SU = ‘ncRNA’ NOT (TI = ‘Review’ OR TI = ‘ Progress' OR TI = ‘Overview’ OR TI = ‘Current situation’),” “FT = ‘Chinese herbal medicine’ AND (SU = ‘lncRNA’ OR SU = ‘miRNA’ OR SU = ‘circRNA’) NOT (TI = ‘Review’ OR TI = ‘ Progress' OR TI = ‘Overview’ OR TI = ‘Current situation’),” “FT = ‘Active ingredient’ AND (SU = ‘lncRNA’ OR SU = ‘miRNA’ OR SU = ‘circRNA’) NOT (TI = ‘Review’ OR TI = ‘Progress’ OR TI = ‘Overview’ OR TI = ‘Current situation’),” “FT = ‘Natural compound’ AND (SU = ‘lncRNA’ OR SU = ‘miRNA’ OR SU = ‘circRNA’) NOT (TI = ‘Review’ OR TI = ‘Progress’ OR TI = ‘Overview’ OR TI = ‘Current situation’),” “FT = 'Natural ingredient’ AND (SU = ‘lncRNA’ OR SU = ‘miRNA’ OR SU = ‘circRNA’) NOT (TI = ‘Review’ OR TI = ‘Progress’ OR TI = ‘Overview’ OR TI = ‘Current situation’),” “FT = ‘Traditional Chinese medicine extract’ AND (SU = ‘lncRNA’ OR SU = ‘miRNA’ OR SU = ‘circRNA’) NOT (TI = ‘Review’ OR TI = ‘Progress’ OR TI = ‘Overview’ OR TI = ‘Current situation’),” or “FT = ‘Traditional Chinese medicine’ AND (SU = ‘lncRNA’ OR SU = ‘miRNA’ OR SU = ‘circRNA’) NOT (TI = ‘Review’ OR TI = ‘Progress’ OR TI = ‘Overview’ OR TI = ‘Current situation’).” “FT” means full text; “SU” means subject; “TI” means title. Articles included in “Guide to Core Journals of China,” “Chinese Science Citation Database” and “Chemical Abstracts” simultaneously, were selected to ensure high quality of literature.

According to the above searching method, English and Chinese original articles related to bioactive ingredients of CHMs and any ncRNA (miRNA, lncRNA, or circRNA) were selected manually.

## Pro-apoptosis Effects of CHMs

Apoptosis is programmed cell death, which is a normal physiological process of cells. Imbalance of apoptosis is closely associated with various diseases, particularly cancer. Proteins that inhibit apoptosis are over-expressed in various cancers, and are considered to be related to tumorigenesis and chemotherapy resistance (Mohamed et al., 2017); therefore, the induction of apoptosis is a promising method for cancer management (Fulda and Vucic, 2012). Recently, several bioactive ingredients of CHMs have been reported to promote apoptosis by targeting miRNA, lncRNA, or ceRNA crosstalk, indicating their potential as complementary therapies for cancer.

### Berberine

Berberine [BBR, 9,10-Dimethoxy-2,3-(methylenedioxy)-7,8,13,13a-Tetrahydroberbinium; Chem. 1] is an isoquinoline alkaloid extracted from the roots of several species including: *Coptis chinensis* Franch., *Berberis soulieana* Schneid., *Berberis poiretii* Schneid., *Berberis vernae* Schneid., *Berberis wilsoniae* Hemsl., and *Platycladus orientalis* (Linn.) Franco. These herbs are considered to have antipyretic and detoxification effects, based on the theory of traditional Chinese medicine (TCM), and were mainly used to treat diseases of the digestive and urinary systems, such as diarrhea, ulcer, jaundice, and urinary infection, as well as dermatological diseases, including eczema. Newly reported researches (Luo et al., 2014; Chai et al., 2018) have found the anti-cancer activity of two extracts from *Coptis chinensis* Franch. one of which is BBR. It's indicated that BBR affects apoptotic pathways in various cancers through regulation of multiple miRNAs. For example, BBR exerts significantly protective effects in multiple myeloma (MM) through targeting several miRNAs. BBR down-regulates the expression of miR-99a~125b, miR-17~92, miR-106~25 (Feng et al., 2015), and miR-21 (Luo et al., 2014), thereby influencing the P53, ERB, and MAPK signaling pathways, leading to acceleration of apoptosis and growth inhibition. Moreover, BBR suppresses MM cell viability through down-regulating miR-19a/92a expression (Yin et al., 2018). Further, BBR can up regulate miR-23a in hepatocellular carcinoma (HCC) (Wang N. et al., 2014), as well as miR-152, miR-429, and miR-29a in colorectal cancer (Huang et al., 2017) in a P53-dependent manner. By inducing NEK6 inhibition and transcriptional activation of the P53-associated tumor suppressor genes, P21 and GADD45α, BBR induces cell death, G2/M cell cycle arrest, and tumor growth suppression in HCC cells; in contrast, miR-23a inhibition can attenuate these BBR-mediated functions (Wang N. et al., 2014). Besides, BBR also regulates cell cycle and inhibits cell proliferation in melanoma A375 cells through promoting miR-34a, miR-154, miR-26a, and miR-124 expression, as well as suppressing target genes CDK4, CyclinD1, CyclinE, and CDK2 (Yang L. H. et al., 2016).

Additionally, BBR can enhance cellular sensitivity to chemotherapeutic drugs, helping to address the problem of drug resistance. In ovarian and gastric cancers, BBR can enhance cisplatin sensitivity by regulating the expression of miR-93 (Chen et al., 2015) and miR-203 (You et al., 2016), respectively, thus inducing apoptosis. Furthermore, there is evidence that BBR can function together with other compound. It has a synergistic antiproliferative effect on colorectal cancer when combined with NVP-AUY922. The potential mechanism underlying this phenomenon was reported to be suppression of CDK4 and induction of miR-296-5p-mediated inhibition of Pin1-β-catenin-cyclin D1 signaling, resulting in cell growth arrest (Su et al., 2015).

Interestingly, BBR also has protective effects against obesity, steatotic liver and insulin resistance. It inhibits cell viability, cell differentiation, and triglyceride content in a dose- and time-dependent manner, through marked induction of miR-27a and miR-27b; while miR-27a and miR-27b inhibitors can counteract this repressive function of BBR (Wu et al., 2016). Steatotic liver results from disordered lipid metabolism, where lncRNA MRAK052686, NRF2 (Yuan et al., 2015), and miR-373 (Li C. H. et al., 2018) are down-regulated. BBR can reverse the abnormal expression of these genes in steatotic liver, thereby inhibiting the AKT-mTOR-S6K signaling pathway and preventing the development of hepatic steatosis (Li C. H. et al., 2018). In addition, through downregulating miR-29a-3p in insulin resistant HepG2 cells, BBR can increase the mRNA and protein expression of IRS1, leading to regulation of the insulin receptor signaling pathway protein (Mao et al., 2018).

Based on the above results, it is clear that BBR possesses different pharmacological effects via targeting miRNAs. Particularly, it shows obvious advantages for treatment of MM and digestive cancers, mainly through its activities in promotion of apoptosis and inhibition of cell growth. More importantly, its direct anticancer properties are strongly associated with P53 signaling. Therefore, BBR presents as promise for potential future use in cancer treatment.

### Artesunate

Artesunate (ART, dihydroartemisinin-12-alpha-succinate; Chem. 2) is a sesquiterpene lactone extracted from the leafy portions of the Chinese herb, *Artemisia annua* L. It's the semisynthetic derivative of artemisinin which is widely known to be a natural antimalarial medicine (Tu, 2016). Recently, the function of ART in cancer therapy, by targeting lncRNA and promoting cell apoptosis, was newly identified. The lncRNA, UCA1, is up-regulated in prostate cancer tissues and positively correlated with poor prognosis (Zhou Y. et al., 2017). ART significantly decreased the expression of lncRNA UCA1, thereby regulating the downstream miR-184/BCL-2 axis, inducing apoptosis, and inhibiting metastatic ability. Furthermore, these protective effects could be reversed by overexpression of lncRNA UCA1, indicating that it is a target of ART (Zhou Y. et al., 2017). Hence, ART exhibits anticancer properties through regulating the ceRNA crosstalk of the lncRNA UCA1/miR-184/BCL-2 axis in prostate cancer. Nevertheless, additional evidence to support these findings is lacking and the stability of this regulatory network requires validation.

### Triptolide/Triptonide

Triptolide (TP, (3bs,4as,5as,6r,6ar,7as,7bs,8as,8bs)-6-hydroxy-6a-isopropyl-8b-methyl-3b,4,4a,6,6a,7a,7b,8b,9,10-decahydrotrisoxireno[6,7:8a,9:4b,5]phenanthro[1,2-c]furan-1(3h)-one; Chem. 3) and Triptonide (TN,(3bS,4aS,5aS,6aS,7aS,7bS,8aS,8bS)-6a-isopropyl-8b-methyl-3b,4,4a,7a,7b,8b,9,10-octahydrotrisoxireno[6,7:8a,9:4b,5]phenanthro[1,2-c]furan-1,6(3H,6aH)-dione; Chem. 4) are both diterpene lactone components originated from *Tripterygium wilfordii* Hook. f. (TwHf) that has traditionally been used for treatment of rheumatoid arthritis (RA). A recent study has revealed that TwHf exerts its anti-rheumatic effects through regulation of miR-146a, which is over-expressed in patients with RA and negatively correlated with prognosis. TwHf treatment could significantly decrease miR-146a expression. Moreover, miR-146a could be used as a predictor of patient clinical response to TwHf (Chen Z. Z. et al., 2017).

Researches about TP and TN broaden the traditional application and generate new pharmacological effect for cancer treatment. TP has been found to exert anticancer activities in lung cancer. It can induce apoptosis and suppress proliferation through inhibiting miR-21 and increasing expression of phosphatase and tensin homolog (PTEN) protein in non-small cell lung cancer. Moreover, miR-21 upregulation could reverse the effect of TP on cell viability and PTEN (Li et al., 2016). Furthermore, TP treatment is also reported to regulate 227 miRNAs and markedly decrease the migration, invasion, and metastasis of lung cancer cells (Reno et al., 2015). In addition, TP can promote apoptosis and suppress cell proliferation in hepatocellular carcinoma, potentially via inhibition of miR-17-92 and miR-106b-25, in a c-MYC-dependent manner, leading to an increase of BIM, PTEN, and P21 levels (Li S. G. et al., 2018).

However, TN shows significant therapeutic advantages in human nasopharyngeal carcinoma (NPC). It promotes NPC apoptosis and cell cycle arrest, as well as inhibition of cell migration and invasion, without toxicity to nasopharyngeal epithelial cells. This anti-cancer activity is attributed to suppression of lncRNA THOR, followed by downregulation of IGF2BP1 mRNA targets involving Myc, IGF2, and Gli1. Furthermore, lncRNA THOR knockout enhances the protection of TN on NPC; while lncRNA THOR overexpression reverses TN-induced treatment in cells. *In vivo*, TN administration also obviously impedes subcutaneous NPC xenograft growth in mice. Similarly, lncRNA THOR knockout inhibits xenograft growth (Wang et al., 2019).

Therefore, TP and TN are attractive candidate chemotherapeutic agents against the above cancers. With regard to TP, PTEN is an important target; while TN possesses anti-cancer activity *in vitro* and *in vivo* through regulating lncRNA THOR/IGF2BP1 signaling. Nevertheless, as the extracts from TwHf with general toxicity (Chen et al., 2008; Luo et al., 2018), the effectiveness and safety of TP and TN require additional confirmation.

### Ailanthone

Ailanthone (AIL, Picrasa-3,13(21)-diene-2,16-dione, 11,20-epoxy-1,11,12-trihydroxy-, (1-beta,11-beta,12-alpha)-; Chem. 5) is a water-soluble quassinoid extracted from the root bark of *Ailanthus altissima* (Mill.) Swingle. Traditionally, the root bark was used for improvement of itching, bleeding and diarrhea in TCM theory. However, AIL has been newly found to possess anti-tumor activity in different tumors (Chen Y. et al., 2017; Peng et al., 2017; Yang P. et al., 2018). Among those, inhibitory effect of human vestibular schwannomas (VSs) induced by AIL is correlated with miRNA. A research has demonstrated that AIL cleaves caspase 3 and caspase 9, promotes Beclin-1, LC3-II accumulation, and decreases p62, cyclin D1 expression, thus increasing apoptotic cell rate. The upstream mechanism may be suppression of miR-21, and the Ras/Raf/MEK/ERK and mTOR pathways, leading to apoptosis and autophagy in AIL-treated cells. In addition, miR-21 overexpression can attenuate the regulation of AIL on Ras/Raf/MEK/ERK and mTOR pathways, as well as apoptosis and autophagy, indicating miR-21 can be the treatment target of AIL in VSs (Yang P. et al., 2018).

### Cordycepin

Cordycepin (COR, 9-(beta-D-3′-Deoxyribofuranosyl)adenine; Chem. 6) is the main bioactive ingredient of *Cordyceps militaris*, a precious CHM. The medicinal herb has immunity-strengthening effect, and has been already used as a health care product in clinical practice. Modern researches broaden the application of COR in various cancers (Wang et al., 2016; Liang et al., 2017; Yu X. et al., 2017). Specifically, treatment of COR for renal cell carcinoma (RCC) is attributed to regulation of miR-21 and PTEN phosphatase. It's indicated that COR down-regulates miR-21 expression and Akt phosphorylation, yet promotes PTEN phosphatase in RCC Caki-1 cells, resulting in induction of apoptotic cell death and suppression of cell migration. Furthermore, miR-21 mimic or PTEN siRNA can markedly abolish the above effects induced by COR (Yang et al., 2017). Therefore, it's confirmed that COR possesses pro-apoptosis and anti-migration function through regulating miR-21/PTEN axis.

In addition, soya-cerebroside (Chem 7), another extracts from *Cordyceps militaris* is demonstrated to be anti-inflammatory for osteoarthritis (OA). It suppresses AMPK and AKT signaling pathways, and then promotes miR-432 expression in OA synovial fibroblasts, leading to inhibition of monocyte chemoattractant protein-1 (MCP-1), monocyte migration and infiltration, as well as cartilage degradation (Liu S. C. et al., 2017). As a result, soya-cerebroside exerts protective effect for OA partially via regulating miR-432, MCP-1, AMPK and AKT pathways; while in this study a clear functional relationships among those factors are not reported.

### Tubeimoside I

TubeimosideI (TBMSI, nosyl]-β-D-glucopyranosyl]oxy]-2,23-dihydroxy-,28-(O-β-D-xylopyranosyl-(1 → 3)-O-6-deoxy-α-L-mannopyranosyl-(1 → 2)-α-L-arabinopyranosyl)ester, intramol. ester, [2β,3β(S),4α]- Tubeimoside TUBEIMOSIDE A(P); Chem. 8) is the main triterpenoid saponin originated from *Bolbostemma paniculatum* (Maxim) Franquet. which has detoxification and detumescent activities. Recent studies have revealed the pharmacological action of TBMS1 as a potential anti-cancer agent (Wang et al., 2011; Gu et al., 2016). A research demonstrated that TBMS1 can promote apoptosis, and attenuate migration, invasion of non-small cell lung cancer cells. The underlying mechanism is attributed to upregulation of miR-126-5p, followed by inactivation of VEGF-A/VEGFR-2/ERK signaling pathway. MiR-126-5p inhibitor can reverse the downregulated VEGF-A and VEGFR-2 induced by TBMS1 treatment; moreover, both miR-126-5p inhibitor, and VEGF-A, VEGFR-2 overexpression upregulate the mRNA expression and phosphorylation of MEK1 and ERK. Significantly, apoptosis, migration and invasion of TBMS1-treated cells can be reversed by either miR-126-5p inhibitor or ERK activator (Shi et al., 2017). From the above results, it can be concluded that miR-126-5p/VEGF-A/VEGFR-2/ERK signaling is the protective pathway of TBMS1 for cancer therapy.

### Oridonin

Oridonin (ORI, (14R)-7-alpha,20-Epoxy-1-alpha,6-beta,7,14-tetrahydroxykaur-16-en-15-one, Chem. 9) is a ent-kaurane diterpenoid compound mainly originated from *Rabdosia rubescens* (Hemsl.) Hara. Traditionally, the herb was convinced to have the effect of detoxification, circulation promotion and pain relief in China. Currently, ORI is illustrated to participate in the treatment of several tumors via different regulatory pathways. It's reported that human laryngeal cancer cell is accelerated to apoptosis after ORI treatment through inhibiting EGFR signaling. Similarly, EGFR suppression increased ORI-induced apoptosis by the promotion of oxidative stress, and activation of intrinsic and extrinsic apoptotic pathways (Kang et al., 2010). Moreover, 105 miRNAs are involved in the regulation of ORI-treated pancreatic cancer (Gui et al., 2015). Therefore, it's possible that miRNAs involve in the anti-cancer activity of ORI; however, whether EGFR is the downstream target of miRNAs deserves more researches.

## Anti-proliferation and Anti-migration Effects of CHMs

Abnormal cell proliferation is involved in the pathogenesis of many diseases. In particular, the proliferation and invasion of cancer cells are primary contributors to poor patient outcomes (Gao et al., 2017). In addition, asthma is also associated with the cell proliferation and migration in airway smooth muscle (Zhao et al., 2016). Hence, suppression of cell proliferation and migration are critical methods for treatment of these diseases. Excitingly, some bioactive ingredients of CHMs have been found to inhibit the proliferation and migration of both cancer and asthma cells through targeting miRNA, lncRNA, or ceRNA crosstalk.

### Curcumin

Curcumin (CUR, (1E,6E)-1,7-Bis(4-hydroxy-3-methoxyphenyl)hepta-1,6-diene-3,5-dione; Chem. 10) is a phenolic compound extracted from *Curcuma longa* L., which was traditionally used as painkiller in rheumatism and other bone and joint diseases. Recent studies have found that CUR can also act as an anticancer agent, via miRNA and lncRNA targets. CUR inhibits miR-208 and activates expression of the cell cycle suppressor, CDKN1A, resulting in dose-dependent suppression of prostate cancer cell proliferation (Guo H. et al., 2015). Further, CUR can significantly increase miR-143 and decrease PGK1 expression, while ectopic expression of FOXD3 can enhance the regulatory effect of CUR on miR-143, thereby inhibiting the proliferation and migration of prostate cancer cells (Cao et al., 2017). Further studies reveal that CUR also acts on human prostate cancer stem cells (HuPCaSC). CUR treatment increases the expression of miR-145 and decreases levels of lncRNA-ROR, the cell cycle proteins CCND1, CDK4, and the stem cell markers OCT4, CD44, and CD133. The tumorigenicity of these cells is thereby significantly reduced through inhibition of their proliferation, invasion, and cell cycle arrest (Liu T. et al., 2017). Moreover, expression levels of miR-770-5p and miR-1247 in the DLK1–DIO3 imprinted gene cluster were significantly up-regulated, leading to suppression of HuPCaSC proliferation and invasion *in vitro* (Zhang et al., 2018a). CUR also promotes the expression of miR-98 in lung cancer, thus inhibiting cell growth and migration (Liu W. L. et al., 2017). By reducing miR-186^*^ expression, it induces apoptosis and decreases cell viability in lung cancer cells as well (Tang et al., 2010). Furthermore, CUR inhibits both proliferation and accelerates apoptosis in bladder, gastric, non-small cell lung, pancreatic cancers, and hepatic carcinoma via the up-regulation of miR-203 (Saini et al., 2011), miR-33b (Sun et al., 2016), miR-192-5p (Jin et al., 2015), miR-7 (Ma et al., 2014), and lncRNA AK125910 (Guo Y. et al., 2015), respectively.

CUR has also been reported to increase the sensitivity of non-small-cell lung cancer (Lu et al., 2017), breast cancer (Zhou S. et al., 2017), and nasopharyngeal carcinoma (Wang Q. et al., 2014) to chemotherapy drugs by targeting ncRNAs including miR-30c, miR-29b-1-5p, and lncRNA AK294004, respectively, along with their downstream genes. Moreover, CUR can exert synergistic effects in combination with other compounds, to suppress cell proliferation and invasion and induce apoptosis in glioblastoma (Wu et al., 2015), breast cancer (Guo et al., 2013), and hepatocellular carcinoma (Zhang S. et al., 2017). In glioblastoma, miR-378 was found to promote the anticancer effect of CUR by regulating p38 expression, demonstrating the mutual interaction of miRNA and CUR (Li et al., 2017). Furthermore, CUR is reported to exert anti-inflammatory effects (Ma F. et al., 2017) and to inhibit adipogenic differentiation (Tian L. et al., 2017).

Notably, as liposome technology is a good method for targeting drug delivery system that can solve the solubility problems of poorly soluble drugs (Allen and Cullis, 2004). A research has used this technology to produce CUR-loaded liposome, increasing solubility and oral bioavailability of CUR, as well as reducing first pass effect of hepar. This drug combination can also promote sensitivity of breast cancer cells to chemotherapy, through regulating different miRNAs of miR-29b-1-5p, miR-29b-3p, miR-6068, miR-6790-5p, and miR-4417, as well as their target genes involving DDIT4, EPAS1, VEGFA, RPS14, and DCDC2 (Zhou et al., 2018).

These data demonstrate that CUR can suppress cell proliferation, growth, and metastasis in various cancers by targeting ncRNAs. In particular, CUR has obvious advantages for the treatment of prostate cancer through its regulation of cancer and cancer stem cells. Moreover, the synergistic effects of CUR with other chemotherapies provide new alternative strategies to address drug resistance. Excitingly, structural improvement of CUR not only ensures its anti-cancer effect, but also promotes the bioavailability.

### Shikonin

Shikonin (SHK, 5,8-dihydroxy-2-((1R)-1-hydroxy-4-methyl-3-penten-1-yl)-1,4-naphthalen-edione; Chem. 11) is a naphthoquinone derivative compound. SHK is extracted from the root of the natural herbal medicine, *Lithospermum erythrorhizon* Sieb. et Zucc. This plant was generally used to treat rash, pox, measles, and urticaria in TCM. Modern studies have discovered broader applications for this compound in cancer, by revealing its anti-proliferation function, which is reported to be associated with targeting of miRNAs. SHK can inhibit proliferation and promote apoptosis by modulating the miR-106b/PTEN/AKT/mTOR signaling pathway in endometrioid endometrial cancer (Huang and Hu, 2018). Moreover, SHK inhibits the proliferation of breast cancer cells through down-regulation of tumor-derived exosomal miR-128 (Wei et al., 2016). In addition, the anticancer activity of SHK in glioblastoma is enhanced by miR-143 by reducing the expression of the anti-apoptosis regulator, BAG3, which is a functional target of miR-143 (Liu et al., 2015).

Overall, the regulatory relationships between SHK and miRNAs are mutual. SHK could target miR-106b and miR-128 in endometrioid endometrial cancer and breast cancer to prevent cell proliferation. Further, miR-143 expression influences the anticancer activity of SHK in glioblastoma. Finally, the results reviewed above demonstrate that the anti-proliferation activity of SHK in cancers can be attributed to its interactions with miRNAs.

### Paeoniflorin

Paeoniflorin[PF,5b-((Benzoyloxy)methyl)tetrahydro-5-hydroxy-2-methyl-2,5-methano-lH-3,4-dioxacyclobuta(cd)pentalen-1a(2H)-yl-beta-D-glucopyranoside; Chem. 12] is the main active ingredient of *Paeonia lactiflora* Pall., which was commonly used to regulate blood circulation and relieve pain in TCM theory. Recent investigations have revealed roles for PF in vasodilation (Goto et al., 1996), anti-inflammation (Chen et al., 2013; Hu et al., 2018), microcirculation improvement (Zhou et al., 2011), anti-oxidation (Chen et al., 2011), and anti-cancer (Wang et al., 2012) activities. Specifically, PF exhibits protective activity in glioma via suppression of cell proliferation and promotion of apoptosis. The potential underlying mechanism may involve upregulation of miR-16 and downregulation of matrix metalloproteinase-9 (MMP-9), which are differentially expressed in glioma tissues and cells compared with healthy controls (Li W. et al., 2015). This result lays the foundation for treatment of cancer using PF; however, supporting evidence is insufficient and more investigations are needed.

### Honokiol

Honokiol (HNK, 5,3′-Diallyl-2,4′-dihydroxybiphenyl; Chem. 13) is a bioactive polyphenol isolated from *Magnolia grandiflora*. Although the flower was traditionally valued as ornamental, it contains the phenolic ingredient, HNK, which has been shown to have antimicrobial activity (Clark et al., 1981). A recent study discovered that HNK has anti-tumor activity; it can markedly inhibit the growth, invasion, and migration of breast cancer cells, and breast-tumor-xenograft growth induced by leptin. HNK promotes the expression of miR-34a, and inhibits WNT1-MTA1-β-catenin signaling, through suppression of STAT3 phosphorylation and recruitment of STAT3 to the promoter of miR-34a (Avtanski et al., 2015). Hence, HNK has demonstrated a protective effect on breast cancer in a diet-induced-obese mouse model with high leptin levels and could serve as a new endocrine therapy drug for patients with obesity-related breast cancer accompanied by negative estrogen and progesterone receptors; however, the research described above was limited to animal experiments, and further evidence in humans is required, thus clinical trials are warranted to further investigate HNK.

### Schisandrin B

SchisandrinB (SchB, 7-dimethyl-ethoxy-stereoisomer;benzo(3,4)cycloocta(1,2-f)(1,3)benzodioxole,5,6,7,8-tetrahydro-1,2,3,13-tetram; Chem. 14) is a type of lignan, extracted from *Schisandra sphenanthera* Rehd. et Wils. The original fruit was commonly used to relieve symptoms of cough, gasp, abnormal sweating, nocturnal emission, thirst, and palpitations, under TCM theory. Although it was widely used to treat various diseases in ancient China, its specific target and underlying mechanism of action were unclear. A recent study of Sch B provided information about the involvement of ncRNA. Sch B may increase the expression of miR-150 and subsequently reduce levels of the lncRNA BCYRN1 in airway smooth muscle cells (ASMCs) of asthmatic rats. By regulating these two ncRNAs, Sch B suppresses the proliferation, viability, and migration of ASMCs; therefore, the study generated evidence that partially explains the mechanism underlying the activity of Sch B against asthma (Zhang X. Y. et al., 2017). Moreover, Sch B can mediate ceRNA crosstalk between miR-150 and lncRNA BCYRN1, further establishing an miR-150/lncRNA BCYRN1/cell proliferation axis; however, as a new regulatory mechanism influencing asthma, the stability of the ceRNA crosstalk requires further investigation.

### Resveratrol

Resveratrol (RES, 3,4′,5-trihydroxystilbene; Chem. 15) is a natural phenol stilbenoid that is mainly found in food, including the skin of grapes and blueberries, and several CHMs, including *Morus alba* L., *Polygonum cuspidatum* Sieb. et Zucc., and *Rubus idaeus* L. It is considered to protect individuals from cardiovascular diseases, as well as dietary and metabolic diseases (Bradamante et al., 2004; Baur et al., 2006; Lagouge et al., 2006). Recently, its anticancer properties have also been evaluated by researchers and RES has been used as a dietary supplement (Garvin et al., 2006; Kalra et al., 2008; Roy et al., 2009). RES can down-regulate the lncRNA, MALAT1, and up-regulate miR-200c, as well as inhibiting WNT/β-catenin signaling, leading to suppression of cell invasion, metastasis, and migration in colorectal cancer (Ji et al., 2013; Karimi Dermani et al., 2017). Moreover, by significantly decreasing oncogenic miR-221 and regulating NF-κB and TFG, RES exerts inhibitory effects on melanoma cells, both *in vitro* and *in vivo* (Wu and Cui, 2017). In glioma, RES inhibits cell proliferation, arrests the cell cycle in S phase, and induces apoptosis *in vitro*, through down-regulation of miR-21, miR-30a-5p, and miR-19, as well as regulating their targets, including P53, PTEN, EGFR, STAT3, COX-2, NF-κB, and the PI3K/AKT/mTOR pathway (Wang G. et al., 2015).

RES also has anti-inflammatory effects. It can reduce expression of miR-155 and promote that of its target gene, suppressor of cytokine signaling 1 (SOCS1), leading to subsequent inhibition of the inflammatory factors, TNF-α, IL-6, MAPKs, and STAT1/STAT3 (Ma C. et al., 2017). Interestingly, by increasing miR-663 expression, RES down-regulates miR-155, thus acting as both an anti-inflammatory and an anticancer agent (Tili et al., 2010). Furthermore, RES exhibits neuroprotective effects. It promotes miR-96 and inhibits its target gene, BAX, resulting in prevention of oxygen/glucose deprivation/re-oxygenation-induced apoptosis and brain damage, while this protective function can be reversed by miR-96 inhibitor (Bian et al., 2017). In Alzheimer's disease, RES also improves long-term memory formation and induction of long-term potentiation of hippocampus CA1 neurons, through down-regulation of miR-134 and miR-124, and up-regulation of CREB and BDNF (Zhao et al., 2013). Therefore, RES is a potential therapeutic agent against cancers, cerebral ischemia, Alzheimer's disease, and other inflammatory conditions.

### Soybean Isoflavones

Soybean isoflavones (SIF, 3-phenyl-4h-1-benzopyran-4-one; Chem. 16) are extracted from *Glycine max* (Linn.) Merr. They act as phytoestrogens in mammals and have been used as dietary supplements. SIF are associated with breast cancer (Douglas et al., 2013; Takagi et al., 2015). Recently, they have also been demonstrated to suppress cell growth and invasion in prostate cancer. A potential mechanism underlying the anti-prostate cancer activity of SIF is its promotion of miR-29a and miR-1256, leading to down-regulation of TRIM68 and PGK-1 by inhibiting methylation of the miR-29a and miR-1256 promoters (Li et al., 2012). Nevertheless, as a controversial ingredient with weak estrogen-like properties, the influence of SIF on hormone-receptor-positive cancers has caused widespread concern. Therefore, research is needed to determine the effectiveness and safety of SIF in the context of different cancers.

### Matrine

Matrine (MAT, (7aS,13aR,13bR,13cS)-Dodecahydro-1H,5H,10H-dipyrido[2,1-f:3′,2′,1′-ij](Memczak et al., 2013; Song et al., 2017)naphthyridin-10-one; Chem. 17) is the main alkaloid extract from *Sophora flavescens Ait* which was commonly used for diseases of dysentery, eczema and jaundice in China. Modern pharmacological research shows that MAT has protective activity in melanoma, as evidenced by inhibition of proliferation and invasion, and promotion of apoptosis in melanoma cell lines. By downregulating miR-19b-3p expression, MAT increases the protein and mRNA expression of PTEN, a direct target of miR-19b-3p. Similarly, miR-19b-3p downregulation can imitate the effect of MAT; while PTEN silencing reverses the protection induced by MAT (Wei et al., 2018). As a result, MAT can exert anti-cancer activity in melanoma via regulating miR-19b-3p/PTEN axis.

### Corylin

Corylin (CL, 3-(2,2-dimethylchromen-6-yl)-7-hydroxychromen-4-one; Chem. 18) is the flavonoid compound extracted from *Psoralea corylifolia* Linn. In TCM practice, *Psoralea corylifolia* Linn. was often used for degenerative bone and joint diseases. Newly reported studies have revealed its application in inflammation (Kim et al., 2016; Hung et al., 2017) and cancer (Chen et al., 2018). The anti-cancer activity induced by CL is related to upregulation of tumor suppressor lncRNA GAS5 and its downstream anticancer pathways activation. As a result, the proliferation, migration, and invasiveness, as well as epithelial-mesenchymal transition are all inhibited in hepatocellular carcinoma cells. Moreover, lncRNA GAS5 silencing can attenuate the above inhibitory effect of CL. In an animal experiment, CL is observed to obviously retard tumor growth as well, with no significant physiological toxicity (Chen et al., 2018). Taken together, lncRNA GAS5 may act as the treatment target of CL in hepatocellular carcinoma; however, specific downstream gene of lncRNA GAS5 still needs further study.

## Anti-inflammatory Effects of CHMs

Inflammation is a common pathological process involved in many diseases, including coronary heart disease, inflammatory bowel disease, myocarditis, asthma, and neuroinflammatory disorder (Harrington, 2017; Robinson et al., 2017; Mahajan et al., 2018); however, both non-steroidal anti-inflammatory drugs and immunosuppressive agents have clear side effects (Shah and Gecys, 2006; Ahmad et al., 2010). Consequently, safe and effective anti-inflammatory drugs for the treatment of the basic pathologies underlying the above diseases are still needed. Several bioactive ingredients of CHMs are reported to target miRNA or ceRNA crosstalk, thereby exerting anti-inflammatory effects.

### Tanshinone IIA

Tanshinone IIA (Tan IIA, Phenanthro [1, 2-b]furan-10, 11-dione, 6, 7, 8, 9-tetrahydro-1, 6, 6-trimethyl; Chem. 19) is a lipophilic diterpenoid extracted from the root of *Salvia miltiorrhiza* Bge. Under TCM theory, the original herb is considered to promote blood circulation. Recent studies have illustrated that Tan IIA has cardioprotective activity (Shang et al., 2012; Feng et al., 2016) and injection of sodium Tan IIA sulfonate has been widely used as an adjunctive therapy for cardiovascular diseases in China (Yu M. L. et al., 2018). A potential mechanism underlying its inhibition of inflammation (Pan et al., 2017; Cheng et al., 2018), and an upstream regulator, is miRNA. Tan IIA can reduce the expression levels of cytokines, chemokines, and acute-phase proteins, including TLR4, MyD88, GM-CSF, sICAM-1, CXCl-1, and MIP-1α. Moreover, it significantly inhibits the mRNA expression levels of IL-1β, TNF-α, and COX-2, thereby suppressing lipopolysaccharides (LPS) -induced activation of the TLR4-NF-κB pathway. Furthermore, expression of miR-155, miR-147, miR-184, miR-29b, and miR-34c is also reduced by Tan IIA, and these may be upstream regulators in anti-inflammation processes (Fan et al., 2016). In addition, by down-regulation of miR-146b and miR-155, Tan IIA significantly reduces the levels of inflammatory factors, including CRP, ox-LDL, IL-1β, IL-6, IL-12, TNF-α, CCL-2, CD40, and MMP-2, thereby exerting protective functions in atherosclerosis induced by *Porphyromonas gingivalis* (Xuan et al., 2017).

Another study indicated that Tan IIA can also inhibit apoptosis caused by hypoxia. Through increasing miR-133 expression and activating the stress-induced protein kinase, MAPK ERK1/2, Tan IIA enhances resistance to hypoxic exposure in neonatal cardiomyocytes (Zhang et al., 2012). Treatment with Tan IIA has also been illustrated to reverse the abnormal expression of miR-1, SRF, and MEF2, and participates in suppression of the p38 MAPK signaling pathway, restoring declined I(K1) current density and Kir2.1 and Cx43 protein levels, thus lowering the incidence of arrhythmogenesis and mortality after myocardial infarction, and improving cardiac function (Shan et al., 2009; Zhang et al., 2010). These results provide a partial explanation for the anti-inflammatory and anti-hypoxia activity of Tan IIA via miRNAs in cardiovascular diseases; in particular, miR-155 may be a specific target of Tan IIA in inflammation.

Additionally, an aqueous extract from *Salvia miltiorrhiza* Bge., named magnesium lithospermate B (MLB, magnesium (2R)-3-(3,4-dihydroxyphenyl)-2-[(E)-3-[(2S,3S)-2-(3,4-dihydroxyphenyl)-3-[(2R)-3-(3,4-dihydroxyphenyl)-1-oxido-1-oxopropan-2-yl]oxycarbonyl-7-hydroxy-2,3-dihydro-1-benzofuran-4-yl]prop-2-enoyl]oxypropanoate; Chem. 20), has neuroprotective effect in ischemia/reperfusion (I/R) injury. I/R injury can lead to miR-107 upregulation, glutamate transporter 1 (GLT-1) suppression and glutamate accumulation, increasing neurological deficit score, infarct volume and cellular apoptosis (Yang Z. B. et al., 2014). MLB treatment improves I/R-induced cerebral injury through reversing the abnormal expressions of miR-107, GLT-1 and glutamate (Yang et al., 2015).

The above results may help to throw light on the underlying mechanisms of Tan IIA and MLB for the treatment of cardiovascular and cerebrovascular diseases from the perspective of miRNA; however, it should be noted that the pharmacological action of *Salvia miltiorrhiza* Bge. is not limited to ncRNAs (Zhu et al., 2017).

### Baicalin

Baicalin (BA, 7-D-glucuronic acid-5,6-dihydroxy-flavone; Chem. 21) is a flavone glycoside extracted from *Scutellaria baicalensis* Georgi, which was commonly applied for the treatment of respiratory and digestive diseases in CHM. The traditional treatment effects may be related to regulation of inflammatory responses. TNF-α stimulation promotes the expression of miR-191a, causing downregulation of ZO-1 mRNA and protein. BA pretreatment could reverse the effects of ZO-1 and miR-191a expression induced by TNF-α, leading to improved viability and migration of rat small intestine epithelial cells. Furthermore, knockdown of miR-191a expression significantly increased BAL-induced ZO-1 protein expression, thereby enhancing the protective effect of BA on cell motility (Wang L. et al., 2017). These data suggest that miR-191a may be an upstream target of BA in the treatment of inflammatory bowel disease; moreover, the therapeutic effects of BA can also be influenced by miR-191a.

Also, another research illustrates the proliferative inhibition of mouse embryonic stem cells induced by baicalin. Baicalin suppresses the expression of miR-294, c-jun and c-fos; while miR-294 overexpression could significantly reverse the above effect of baicalin, indicating miR-294 may be the treatment target (Wang J. et al., 2015).

Therefore, BA exerts anti-inflammatory and anti-proliferative effects by targeting miRNAs and emerges as a potential treatment agent for digestive disease. Further, as BA remains one of the most frequently used medicines for the treatment of cough and phlegm, the activity of BA in respiratory disease warrants similar studies.

### Cinnamaldehyde

Cinnamaldehyde (CA, 3-phenylprop-2-enaldehyde; Chem. 22) is a conjugated aromatic aldehyde extracted from the bark of the Chinese herb, *Cinnamomum cassia* Presl. According to TCM theory, the traditional plant can enhance the function of “yang qi” (a substance with excitatory function in TCM) and is often used to relieve symptoms of weakness. Recent studies have broadened the application of this preparation to the treatment of cerebrovascular diseases, ulcerative colitis, and cancer (Zhao et al., 2015; Tian F. et al., 2017; Qu et al., 2018), where it acts by exerting anti-inflammatory or ncRNA regulatory functions. CA improves symptoms of weight loss, disease activity index, and infiltration of inflammatory cells, by decreasing the levels of pro-inflammatory cytokines, including TNF-α, IL-1β, and IL-6, as well as the NLRP3 inflammasome, miR-21, and miR-155, in both colon tissue and macrophages. Moreover, levels of reactive oxygen species were also reduced, along with the phosphorylation of AKT, mTOR, and COX2 proteins. Further experiments revealed similar suppression of IL-1β and IL-6 in response to miR-21 or miR-155 inhibitors, demonstrating that these inflammatory factors are positively regulated by miR-21 or miR-155 (Qu et al., 2018). As a result, CA suppresses the miR-21/miR-155/IL-1β/IL-6 axis to exert its protective function in ulcerative colitis.

CA also has anti-cancer activity through regulation of ceRNA crosstalk and can suppress cell proliferation and induce apoptosis in non-small cell lung cancer. Through upregulation of has-circ-0043256 and ITCH expression, CA inhibits the WNT/β-catenin pathway, while this function can be partially abolished by miR-1252, indicating that miR-1252 may participate in has-circ-0043256-related regulation. Moreover, has-circ-0043256 knockdown can reverse the effects of CA on cells (Tian F. et al., 2017). Consequently, has-circ-0043256/miR-1252/ITCH crosstalk may contribute to the anticancer activity of CA.

### Geniposide

Geniposide (GEN, methyl (1S,4aS,7aS)-7-(hydroxymethyl)-1-[(2S,3R,4S,5S,6R)-3,4,5-trihydroxy-6-(hydroxymethyl)oxan-2-yl]oxy-1,4a,5,7a-tetrahydrocyclopenta[c]pyran-4-carboxylate; Chem. 23) is derived from *Gardenia jasminoides Ellis*, a traditional antipyretic and detoxifying CHM. A recent research reported GEN has effects of anti-inflammatory and cardiomyocyte protection in LPS-injured H9c2 cells. It up-regulates miR-145 expression, inhibits pro-inflammatory factors of IL-6, TNF-α, and MCP-1, and then suppresses the MEK/ERK pathway, thus promoting cell viability and inhibiting apoptosis. Moreover, miR-145 inhibitor could reverse the above protective function induced by GEN pretreatment (Su et al., 2018). Therefore, GEN becomes a potential therapeutic agent for myocarditis in practice by targeting miR-145 and anti-inflammation in cardiomyocyte.

### Carvacrol/Thymol

Carvacrol (Car, 5-Isopropyl-2-methylphenol; Chem 24) and Thymol (Thy, 2-Isopropyl-5-methylphenol; Chem. 25) are isolated from the essential oil of *Origanum vulgare* L. or wild bergamot. They are isomers and belong to monoterpenoid phenol. Traditionally, *Origanum vulgare* L. was applied for the treatment of cold and heatstroke. Bergamot can relieve pain and vomit under TCM theory. A research further expanded the applicable scope of these herbs by studying the two bioactive ingredients. Car/Thy can suppress the allergic inflammation in asthma by regulating miRNAs and inflammatory factors. In chitin-induced model, expression levels of miR-155, miR-146a and miR-21, promotor of pro-inflammatory cytokines, are upregulated. Furthermore, SOCS1 and SHIP1, targets of miR-155 and negative regulators of TLR-mediated inflammation, are demonstrated to be inhibited by chitin. However, Car/Thy treatment can reverse the abnormal expressions of TLR2, TLR4, SOCS1, SHIP1, and miR-155, miR-146a, miR-21 (Khosravi and Erle, 2016). These results preliminarily establish the relationships between anti-inflammation effect of Car/Thy and miRNAs; but the specific target and corresponding regulatory network are not reported regrettably.

### Boswellic Acids

Boswellic acids are extracted from oleo-gum-resin of *Boswellia serrata*, a traditional CHM with promoting blood circulation and pain relief function. Boswellic acids contain different ingredients, among which 3-acetyl-11-keto-β-boswellic acid (AKBA, (3R,4R,6aR,6bS,8aR,11R,12S,14bS)-3-acetyloxy-4,6a,6b,8a,11,12,14b-heptamethyl-14-oxo-1,2,3,4a,5,6,7,8,9,10,11,12,12a,14a-tetradecahydropicene-4-carboxylic acid; Chem. 26) possesses the most potent anti-inflammatory activity (Siddiqui, 2011; Sayed et al., 2018). AKBA can attenuate the behavioral dysfunction in LPS-induced neuroinflammation, similarly with that effect of dexamethasone. Moreover, AKBA lowers expression of miR-155, P-IκB-α, and carbonyl protein, and increases contents of normal cytokine and SOCS-1, resulting in effects of anti-apoptotic and anti-amyloidogenic (Sayed et al., 2018). Therefore, regulation of miR-155 and downstream protein helps to reveal the possible mechanism underneath AKBA's positive role in neuroinflammatory disorders; however, the specific target deserves more verification.

## Anti-atherosclerosis Effects of CHMs

Atherosclerosis is the basic pathology underlying coronary artery disease, cerebral infarction, and other vascular diseases (Pothineni et al., 2017; Li Q. et al., 2018). Medicines with anti-atherosclerosis activities are therefore highly significant for the prevention and treatment of these diseases. Statins are currently the main drugs used against atherosclerosis; however, when taken for long periods of time, they risk impairing liver function and causing muscle lysis, particularly in elderly patients (Guyton, 2006; Ramakumari et al., 2018). Therefore, better drugs with relatively few side effects are needed and CHMs represent a good resource in this context. Recent studies have identified three bioactive ingredients of CHMs as regulators of atherosclerosis through targeting miRNA or lncRNA.

### Sinapic Acid

Sinapic Acid (SA, 3,5-dimethoxy-4-hydroxycinnamic acid; Chem. 27) is the bioactive ingredient isolated from seeds of the Chinese herb, *Sinapis alba* L. The seeds were commonly used to treat cough, phlegm, limb numbness, and chronic abscess. A recent study reported protective effects of SA in atherosclerosis, which helped to partially explain the original application of the seed to treat limb numbness. The lncRNA MALAT1 is significantly up-regulated in rats with diabetic atherosclerosis and low-dose SA treatment can suppress this abnormal expression. Subsequently, pyroptotic death of bone marrow derived macrophages is inhibited, accompanied by decreased expression of ET-1 and IL-1β, and the pyroptotic proteins, ASC, NRLP3, and Caspase-1 (Han et al., 2018). Hence, SA exerts anti-inflammatory activity and prevents pyroptosis, thus exerting anti-atherosclerosis effects, through targeting lncRNA MALAT1; however, the efficacy and safety of SA as a potential treatment agent require verification by additional studies.

### Polydatin

Polydatin (PLD, 3,4,5-trihydroxystilbene-3-beta-monoglucoside; Chem. 28), also known as Piceid, is the bioactive ingredient from *Polygonum cuspidatum* Sieb. et Zucc. This herb was traditionally used for the treatment of jaundice and cough. However, PLD has also been found to exert protective effects against cardiac hypertrophy (Zhang et al., 2015a), insulin resistance, and hepatic steatosis (Zhang et al., 2015b). Further, a recent study has revealed the underlying regulatory action of PLD in atherosclerosis with liver dysfunction. The findings indicated that PLD treatment can markedly lower increased blood glucose, serum ALT, AST, TC, TG, and LDL-C in mice with high-fat diet. Simultaneously, changes in HDL-C, MDA, SOD, and miR-214 were also improved in liver tissue (Zhou et al., 2016). This study indicates that PLD can be therapeutically effective in complex diseases by regulating various factors. In addition, PLD shows great potential as a complement to treatment for statin-induced liver damage via its anti-atherosclerosis and liver protection properties; however, the above study only reported the expression levels of various factors induced by PLD, rather than systematically studying the relationships between miR-214 and its target genes. Therefore, further in-depth investigations are required in the future.

### Ampelopsin

Ampelopsin [(2r,3r)-3,5,7-trihydroxy-2-(3,4,5-trihydroxyphenyl)chroman-4-one; Chem. 29], alsocalled dihydromyricetin (DHM), is the main flavonoid compound from *Ampelopsis grossedentata*. The original herb has effects of detoxification, anti-inflammatory and analgesic, commonly used as a dietary supplement. DHM is now demonstrated to impede atherosclerotic process by regulating endothelial dysfunction (Yang D. et al., 2018), and exert anti-aging effect against neurodegenerative diseases (Kou et al., 2016). It inhibits miR-21 expression and then improves endothelial dysfunction induced by TNF-α, accompanied by suppression of abnormal expression of eNOS, DDAH1, NO, and ADMA, as well as improvement of tube formation and migration. Furthermore, miR-21 blockade can produce similar effects with DHM treatment; while miR-21 overexpression abolishes the above protection. Additionally, improvement of endothelial dysfunction can be reversed by a non-specific NOS inhibitor, indicating DHM ameliorates vascular endothelial function and inhibits atherosclerosis by targeting miR-21-mediated DDAH1/ADMA/NO signal pathway (Yang D. et al., 2018).

Another study reveals that miR-34a is upregulated in D-gal-induced brain aging rats; while DHM management can inhibit the abnormal expression. Moreover, DHM suppresses apoptosis and ameliorates impaired autophagy of neurons in D-gal-injured hippocampus tissue, by up-regulating SIRT1 and down-regulating mTOR signal pathways (Kou et al., 2016). Therefore, DHM possesses anti-aging effect partially through regulating miR-34a-mediated SIRT1-mTOR signal pathway, showing important role for the treatment of neurodegenerative diseases.

From the above results, it can be seen that DHM exerts not only anti-atherosclerosis effect, but also anti-aging function by targeting miRNAs and downstream signaling pathways.

## Anti-infection Effects of CHMs

Antibiotics and antiviral drugs are basic treatments for infectious diseases; however, a deteriorating situation caused by antibiotic abuse, drug-resistance, and viral mutations is shifting the focus of research attention to other therapeutic and complementary drugs for treatment of these conditions (Miyoshi et al., 2015; Jiang et al., 2019). To date, two bioactive ingredients of CHMs have been found to contribute to the treatment of infection through regulation of miRNAs. These substances can protect the human body from pathological damage, although they do not directly induce pathogen resistance.

### Icariine

Icariine [ICA, 2-(4′-methoxyphenyl)-3-rhamnosido-5-hydroxyl-7-glucosido-8-(3′-methyl-2-butylenyl)-4-chromanone; Chem. 30] is the main bioactive flavonoid glucoside extracted from *Epimedium brevicornu* Maxim. Under TCM, this herb was considered to nourish “yang qi” and generally applied for treatment of osteoarticular and reproductive diseases. ICA can suppress osteoclast bone resorption and bone loss, indicating great potential for use as a treatment agent for both aseptic loosening and bacteria-induced bone loss (Zhang et al., 2015; Liu et al., 2016). Specifically, ICA can restore LPS-induced bone loss, without obvious cytotoxicity. This product can down-regulate expression of the osteogenic inhibitor, miR-34c, while it up-regulates levels of the key transcription factor, RUNX2, thereby inducing osteogenic differentiation and mineral nodule formation. Moreover, miR-34c overexpression can reverse these effects of ICA. Additionally, ICA markedly suppresses LPS-induced activation of JNK, p38, and NF-kB pathways, leading to therapeutic effects in diseases causing bacteria-induced bone loss, such as osteomyelitis and septic arthritis (Liu et al., 2016). Interestingly, ICA also exhibits anticancer activity in ovarian cancer via down-regulation of miR-21 expression. This subsequently induces PTEN, RECK, and Caspase-3 activity, while BCL-2 protein expression is inhibited, leading to suppression of cell proliferation and increased apoptosis (Li J. et al., 2015). Based on these findings, miR-34c appears to facilitate the mechanisms underlying the role of ICA in infectious bone loss. Furthermore, the identification of miR-21 suggests a potential new application of ICA in cancer therapy. Therefore, there is promise that additional currently unknown functions of this medicinal herb could be determined by studying ncRNA and related regulatory networks.

### Ginsenosides

Ginsenosides (GS,(3S,5R,8R,9R,10R,14R,17S)-17-(2-hydroxy-6-methylhept-5-en-2-yl)-4,4,8,10,14-pentamethyl-2,3,5,6,7,9,11,12,13,15,16,17-dodecahydro-1H-cyclopenta[a]phenanthren-3-ol; Chem. 31), also referred to as panaxosides, are a class of natural steroid glycosides and triterpene saponins. These products include various active components, such as ginsenoside Re, Rg, Rh, Rb, and Rc. GS are mainly isolated from *Panax ginseng* C. A. Mey., a valuable herb with nourishing effects and a long history of use in ancient China. At present, GS products are not only used to promote health, but also for their activity as immune regulators in many diseases (Jiang Z. et al., 2017; Shin et al., 2018; Yu X. et al., 2018). GS exert a cytoprotective effect, thereby promoting cell viability on avian influenza H9N2/G1 infection. During this process, the expression of miR-15b was up-regulated, while production of IP-10 was markedly inhibited. Furthermore, cytometry and TUNEL analyses indicated that ginsenoside Re prevents apoptosis and DNA damage in human endothelial cells caused by H9N2/G1 (Chan et al., 2011). These results are inconsistent with the traditional concept that GS is only suitable for treatment of sub-optimal health status or chronic diseases and greatly expand the potential for application of GS for treatment of acute infectious diseases in the future.

## Anti-senescence Effects of CHMs

Cell senescence is the irreversible state in which cells undergo cycle arrest responding to various factors (Watanabe et al., 2017). It participates in biological processes involving embryonic development, wound healing and aging, closely relating to organismal aging and diseases, and thus arousing widespread concerns in researchers (Watanabe et al., 2017; de Magalhães and Passos, 2018). Currently, several bioactive ingredients of CHMs have been found to act positive roles in anti-senescence.

### Salidroside

Salidroside [SAL, 2-(4-Hydroxyphenyl)ethyl beta-D-glucopyranoside; Chem. 32] is the main bioactive extract from *Rhodiola rosea* L. with effects of nourishing “yang qi” and promoting blood circulation under TCM theory. Modern pharmacological study further revealed that the medicinal herb not only exerts anti-fatigue ability, but also improves resistance to hypoxia (Li et al., 2014). Moreover, SAL has been supported to possess anti-senescence activity. The potential mechanism is related to regulation of multiple miRNAs expression. Through upregulating let-7c, let-7e, miR-3620, and decreasing expression of miR-411, miR-24-2-5p and miR-485-3p in the aging cells, SAL participates in several pathways involving p53, transcription factor CREB and AKT/mTOR signaling (Zhang J. et al., 2017). As is known that both let-7 and mTOR are aging-related (Marasa et al., 2010; Wu et al., 2013), and the former factor can directly inhibit the expression of the latter (Marcais et al., 2014). Therefore, it's possible that SAL possesses anti-senescence effect by regulating let-7 and mTOR; however, the predicted regulatory relationship requires more validation in the future.

### Phlorizin

Phlorizin (PZ, Phloretin-2′-O-beta-glucoside; Chem. 33) is the main active ingredient of *Acanthopanax senticosus* (Rupr. et Maxim.) Harms which is a traditional CHM with functions.of nourishing and enhancing strength. PZ is convinced to exert effects of anti-fatigue, learning improvement and immune-enhancing (Huang et al., 2011). Researches further reported that PZ can act as a promising agent for skin aging (Zhai et al., 2015; Choi et al., 2016). By promoting epidermal cell proliferation and self-renewal, PZ thickens epidermis to maintain skin structure and resistance to aging. Moreover, PZ increases expression of p63 and proliferating cell nuclear antigen (PCNA), as well as integrin α6, integrin β1 and type IV collagen. Particularly, the mRNA of type IV collagen is also increased and possibly regulated by downregulation of miR-135b (Choi et al., 2016). As a result, miR-135b/type IV collagen axis may be the underlying regulatory mechanism of anti-senescence induced by PZ.

### Osthole

Osthole (Ost, 7-methoxy-8-(3-methyl-2-butenyl)-2H-1-benzopyran-2-one; Chem. 34) is mainly extracted from *Cnidium monnieri* (L.) Cuss. which was commonly used for nourishing “yang qi” and relieving itching in TCM practice. Current pharmacological researches newly revealed that Ost can improve memory, delay senescence and resist cell damage in Alzheimer's disease (AD) (Hu et al., 2013; Zheng et al., 2013). As it's clear that beta-amyloid peptide (Aβ) is the critical pathology of AD, inhibition of Aβ deposition thereby becomes an important treatment strategy for the disease (Wilcock et al., 2009). Ost was reported to enhance cyclic AMP response element-binding protein (CREB) phosphorylation and then inhibit Aβ cytotoxicity on neural cells (Hu et al., 2013). Further mechanism study indicates that it upregulates miR-107, and then promotes cells viability of neuron, resulting in suppression of the protein expression of Aβ and BACE1, as well as LDH (Xiao et al., 2017). Therefore, Ost exertes obvious neuroprotective effect through targeting miR-107 and impeding Aβ deposition, presenting as a potential treatment agent for neurogenic aging and neurodegenerative disease.

## Inhibitory Effects of CHMs on Structural Remodeling

Structural remodeling is an important factor that can impede the normal functions of tissues and organs. It is also the main pathological change during the late stages of various diseases, making poor prognosis and difficult treatment (Bijkerk et al., 2019; Bittencourt et al., 2019; Zhuang et al., 2019). Encouragingly, three CHM ingredients have been demonstrated to exert protective effects on this pathological change through regulation of miRNAs.

### Panax Notoginseng Saponins

Panax notoginseng saponins (PNS, notoginsenoside-fe, 98%; Chem. 35) are a chemical mixture extracted from the root of *Panax notoginseng* (Burk.) F. H. Chen. According to TCM theory, the traditional herb can simultaneously promote blood circulation and prevent bleeding; therefore, it was commonly used to treat coronary artery disease, stroke, gastrointestinal bleeding, irregular menstruation, and bruises. Currently, several PNS preparations, including xuesaitong injections and xuesetong capsules, are widely used to treat cardiovascular diseases (Song et al., 2017). The improvement in cardiac prognosis caused by PNS has been attributed to its regulation of miRNAs and suppression of structural remodeling. PNS was reported to increase expression of the anti-fibrotic factor, miR-29c, which is clearly reduced in mice with isoproterenol-induced myocardial fibrogenesis, leading to downregulation of its target genes: collagen (Col) 1a1, Col1a2, Col3a1, Col5a1, Fbn1, and TGFβ1, thus exerting protective effects against myocardial injury and fibrosis (Liu L. et al., 2017). In addition, PNS has obvious resistance to H_2_O_2_-induced oxidative damage, showing anti-apoptosis activity in vascular endothelial cells (VECs) by suppressing miR-146b-5p expression (Wang J. et al., 2017). Moreover, notoginsenoside R1, one main component of PNS, can delay the process of senescence in VECs by regulating miR-34a/SIRT1/p53 pathway (Lai et al., 2018). As a result, through repairing VECs damages, PNS inhibits vascular pathological process.

PNS also has an active role in tumors complicated by myocardial ischemia where paradoxical treatment strategy existed. PNS and its major components, Rg1, Rb1, and R1, are implicated in tissue-specific regulation of angiogenesis, and can inhibit tumor growth, as well as attenuating myocardial ischemia. The potential underlying mechanism may be the down-regulation of miR-18a and vascular markers (CD34 and vWF) in tumor, with simultaneous up-regulation of these factors in heart (Yang Q. et al., 2014). Notably, by modulating Met/miR-222 axis, and then increasing target genes of tumor suppressor p27 and PTEN expression, PNS selectively inhibits the survival of lewis lung carcinoma cells and attenuates tumor growth in mice (Yang Q. et al., 2016).

Based on the above results, PNS appears to exert its cardioprotective function by preventing fibrosis, improving vascular endothelium, and promoting angiogenesis in the heart. Simultaneously, considering the cardioprotection and anti-tumor effects through targeting miRNAs, PNS is especially suitable for patients with heart disease and tumor.

### Tetrandrine

Tetrandrine (TET, 6,6′,7,12-tetramethoxy-2,2′-dimethyl-1 beta-berbaman; Chem. 36) is a bisbenzylisoquinoline alkaloid extracted from *Stephania tetrandra* S. Moore. The medicinal plant was often used for treatment of rheumatism and edema under the theories of TCM. Notably, the source plant should not be confused with *Radix Aristolochiae Fangchi* which causes nephrotoxicity, despite their similar Chinese names. A recent study identified a new pharmacological effect of TET in treatment of anti-hypertrophic scarring. The underlying mechanism was suggested to be repression of DNA and collagen synthesis in scar-derived fibroblasts (Liu et al., 2001). Furthermore, by upregulating miR-27b and downregulating miR-125b, TET influenced the expression of putative targets, including VEGFC, BCL2L12, COL4A3, and FGFR2, predicted to contribute to several scar and wound healing-related signaling pathways (Ning et al., 2016). Consequently, TET has therapeutic potential for the inhibition of skin tissue hyperplasia after wounding or surgery.

### Leonurine

Leonurine (LEO, 4-Guanidino-n-butyl syringate; Chem. 37) is the alkaloid compound from *Leonurus artemisia* (Laur.) S. Y. Hu F, which was commonly used for gynecological diseases in TCM. Newly reported results have indicated the cardioprotective effect by studying LEO activity, namely anti-atherosclerosis (Jiang T. et al., 2017), anti-oxidation (Gao et al., 2016) and resistant to cardiomyocyte hypertrophy (Lu et al., 2019). LEO treatment can significantly reduce the surface area of hypertrophic cardiomyocytes, decrease the content of atrial natriuretic peptide (ANP), endothelin-1 (ET-1), p38 mitogen-activated protein kinase (p38 MAPK), phosphorylated p38 MAPK (p-p38 MAPK), myocyte enhancer factor 2 and β-myosin heavy chain. Moreover, it also up-regulates the expression levels of α-myosin heavy chain protein and miR-1. Thus, by upregulating miR-1 expression and then inhibiting the activation of p38MAPK signaling pathway, LEO may inhibit AngII-induced cardiomyocyte hypertrophy and structural remodeling (Lu et al., 2019).

## Other Effects of CHMs

Bioactive ingredients of CHMs can exert various protective effects through targeting different miRNA, lncRNA, or circRNA. Besides the above mentioned mechanisms, some ingredients also play positive roles in anti-I/R injury, anti-arrhythmia, recovery of blood-spinal cord barrier, and promotion of cardiac differentiation by targeting lncRNA and miRNA.

### Calycosin/Astragaloside IV

Calycosin (CAL, 7,3′-dihydroxy-4′-methoxyisoflavone; Chem. 38) is a natural phytoestrogen derived from *Astragalus membranaceus* (Fisch.) Bunge. which can nourish “yang qi” and was commonly used for cardiovascular and cerebrovascular diseases in TCM practice. It's indicated that the neuroprotection effect of CAL is related to miRNA. CAL markedly improves the infarcted volume, brain water content, and neurological deficit in cerebral I/R injury rats, by upregulating miR-375, ER-α and Bcl-2, and inhibiting RASD1 expression (Wang Y. et al., 2014). Regrettably, a systematic mechanism of miR-375 and those downstream targets has not been revealed in this study.

Moreover, CAL also possesses positive role in anti-cancer, enriching the pharmacological effect and application of *Astragalus membranaceus* extracts (Tseng et al., 2016; Kong et al., 2018). It's demonstrated that CAL significantly impedes lncRNA EWSAT1 expression in nasopharyngeal carcinoma (NPC), followed by influenced downstream factors and pathways, leading to inhibitory growth. Furthermore, lncRNA EWSAT1 overexpression can reverse CAL-induced effect, indicating lncRNA EWSAT1 act as the specific target of CAL promisingly (Kong et al., 2018).

Additionally, Astragaloside IV (ASIV, 3-O-beta-D-xylopyranosyl-6-O-beta-D-glucopyranosylcycloastragenol; Chem. 39), another bioactive compound of *Astragalus membranaceus* (Fisch.) Bunge., can ameliorate precancerous lesions of gastric carcinoma (PLGC) markedly. It lowers mRNA and protein expressions of LDHA, MCT1, MCT4, HIF-1α, and CD147, as well as increasing TIGAR and p53 content. Furthermore, ASIV treatment promotes miR-34a expression. As a result, ASIV improves abnormal glycolysis and dysplasia possibly via regulation of miR-34a/LDHA pathway (Zhang C. et al., 2018).

Interestingly, the total flavonoids of *Astragalus membranaceus* (Fisch.) Bunge. (TFA) can improve heart function damaged by viral myocarditis. By upregulating the expression of miR-378 and miR-378^*^ in cardiomyocytes infected with coxsackie B3 virus, TFA may inhibit cardiac hypertrophy and improve prognosis (Nagalingam et al., 2013; Wan et al., 2017). Therefore, it can be speculated that the heart protection of TFA is attributed to inhibition of myocardial pathology by regulating miR-378 and miR-378^*^.

From the above results, it can been seen that although CAL, ASIV and TFA are extracted from the same herb, they have different targets and are applicable for distinct diseases, demonstrating the study necessity of identified ingredients and targets from CHMs.

### Paeonol

Paeonol (PAE, 2′-Hydroxy-4′-methoxyacetophenone; Chem. 40) is the main bioactive ingredient of *Paeonia suffruticosa* Andr. and *Cynanchum paniculatum* (Bunge) Kitagawa. The two herbs promoted blood circulation and could be used for cardiovascular diseases in TCM practice. Further pharmacological research shows PAE significantly reduces the incidence of ischemic arrhythmia in rats, including lowered frequency of ventricular premature beat, ventricular tachycardia and ventricular fibrillation. Moreover, it markedly decreases infarct size of myocardium. The potential treatment target is miR-1 which is inhibited by PAE in cardiomyocytes (Zhang and Xiong, 2015). Nevertheless, the above study only reveals the possible regulatory relationship between PAE and miR-1, which needs more verification and further identifies the downstream target gene of miRNA.

### Salvianolic Acid A

Salvianolic acid A (Sal A, (R)-3-(3,4-Dihydroxyphenyl)-2-(((E)-3-(2-((E)-3,4-dihydroxystyryl)-3,4-dihydroxyphenyl) acryloyl)oxy)propanoic acid; Chem. 41) is derived from water-soluble phenolic compound of *Salvia miltiorrhiza* Bge. It has protective effects of anti-IR injury (Jiang et al., 2008; Yang et al., 2011), recovery of neurological function (Yu D. S. et al., 2017) and anti-cancer activities (Chen et al., 2016; Lu et al., 2016), the latter two pharmacological actions of which are attributed to miRNA regulation. It's reported that Sal A significantly increases expression of tight junction proteins and HO-1, and decreases p-caveolin-1 and apoptosis-related proteins, resulting in recovery of blood-spinal cord barrier integrity after spinal cord injury (SCI). Furthermore, HO-1 inhibitor can attenuate the regulation of ZO-1, occluding, and p-caveolin-1 by Sal A. The underlying target and mechanism may be upregulation of miR-101 which promotes expression of nuclear factor erythroid 2-related factor 2 (Nrf2) and HO-1. Conversely, miR-101 inhibitor accelerates the permeability of rat brain microvascular endothelial cells, and the protein of Cul3 by targeting its mRNA. As a result, Sal A improves neurological function after SCI through targeting miR-101/Cul3/Nrf2/HO-1 signaling pathway (Yu D. S. et al., 2017).

Another study indicated that Sal A can also down-regulate the expression of multidrug resistance gene MDR1 in lung cancer, thereby emerging as a new treatment agent for lung cancer resistance. The potential mechanism may be related to up-regulation of 4 miRNA expressions including miR-3686, miR-4708-3p, miR-3667-5p, and miR-4738-3p (Chen et al., 2016). This study attempts to find the upstream target of Sal A against MDR1 from perspective of post-transcriptional regulation; but current result cannot directly confirm the regulatory correlation between the 4 miRNAs and MDR1. Thus, more and deeper experiments are urgently needed in the future.

### Andrographolide

Andrographolide (Andro, 17-hydro-9-dehydro-andrographolide; Chem. 42) is a diterpenoid lactone compound derived from *Andrographis paniculata* (Burm. f.) Nees, a natural anti-bacterial and anti-viral CHM. A recent study further demonstrates that Andro can inhibit hepatoma tumor growth. It promotes the expression of 22 miRNAs, but declines that of other 10 miRNAs in a xenograft mouse tumor model *in vivo*. Among those upregulated miRNAs, miR-222-3p, miR-106b-5p, miR-30b-5p, and miR-23a-5p are confirmed in cell experiments *in vitro*. Functional analysis reveals that those miRNAs are mainly involved in signaling pathways of miRNAs in cancer, MPAKs and focal adhesion. Moreover, 24 target genes involved in the above signaling pathways are illustrated to be consistent with miRNAs expression (Lu et al., 2016). As a result, Andro prevents hepatoma tumor growth partially through regulating miRNA profile; whereas the specific target and underlying mechanism still need deeper study.

### Puerarin

Puerarin (PUE, 7,4'-Dihydroxy-8-C-glucosylisoflavone; Chem. 43) is the main active ingredient extracted from *Radix Puerariae Lobatae* which improved symptoms of fever, neck stiffness, thirst and diarrhea in ancient China. Modern researches reveal the cardiovascular protection of PUE for myocardial infarction (Zhang et al., 2006) and arrhythmia (Zhang et al., 2011). The active effects may be attributed to promotion of cardiac differentiation (Cheng et al., 2013; Wang L. et al., 2014). PUE upregulates expression of caveolin-3, amphiphysin-2 and junctophinlin-2, and then ameliorates myofibril array and sarcomeres formation, accompanied by increased t-tubules development in the embryonic stem cell-derived cardiomyocytes. Moreover, PUE suppresses the upstream regulatory factor of caveolin-3, namely miR-22, indicating miR-22/caveolin-3 axis may be the underlying mechanism of cardiomyogenesis induced by PUE (Wang L. et al., 2014).

## Conclusion

CHM has long been a powerful weapon used by Chinese people to combat disease. Over thousands of years, practitioners of CHM have accumulated a wealth of knowledge which is used to prevent and cure diseases. The theoretical concepts of TCM act as the basis for scientific research into CHM today, including identification of the bioactive ingredients and underlying mechanisms of CHMs that could be of benefit internationally—a gift from the Chinese people to the world (Tu, 2011, 2016). Chinese pharmacologist, Youyou Tu, and her team discovered the highly effective and low-toxicity bioactive ingredient “artemisinin” from *Artemisia annua* L. (also referred to as “qinghao” in Chinese), inspired by, “*A Handbook of Prescriptions for Emergencies”* (written around 317–420 CE), thus making an outstanding contributions to the global treatment of malaria (Tu, 2016). Consequently, we became convinced that study of the bioactive ingredients of CHMs is an effective way to reveal their potential mechanisms of action and further broaden their clinical application.

By conducting the above comprehensive review, we found that bioactive ingredients of CHMs can play positive roles in treatment of cancer, cardiovascular, nervous system, respiratory, digestive, infectious, and senescence-related diseases. Through targeting various miRNAs, lncRNAs, circRNAs, or ceRNA crosstalk, these ingredients exert protective effects, including pro-apoptosis, anti-proliferation and anti-migration, anti-inflammation, anti-atherosclerosis, anti-infection, anti-senescence, and anti-structural remodeling. Some miRNAs, including miR-21, miR-34a, miR-34c, miR-155, miR-29a, miR-203, miR-27b, miR-184, and miR-143, contributed to the treatment mechanisms of more than one bioactive ingredient of CHMs. In particular, miR-21 was identified as targeted and regulated by BBR (Luo et al., 2014), TP (Li et al., 2016), RES (Wang G. et al., 2015), CA (Qu et al., 2018), ICA (Li J. et al., 2015), AIL (Yang P. et al., 2018), Car/Thy (Khosravi and Erle, 2016), DHM (Yang D. et al., 2018), and COR (Yang et al., 2017), especially in its anti-cancer activities, indicating that this miRNA is stably targetable and responsive to the pharmacological effects of various CHMs. Moreover, miR-155 was associated with inflammatory responses and could be inhibited by CUR, RES, Tan IIA, CA, Car/Thy and AKBA, in inflammatory-related diseases (Tili et al., 2010; Fan et al., 2016; Khosravi and Erle, 2016; Ma F. et al., 2017; Xuan et al., 2017; Qu et al., 2018; Sayed et al., 2018). Thus, it is highly likely that miR-155 could represent a new treatment target for anti-inflammation. In addition, three complex ceRNA crosstalk networks were discovered to function in the therapeutic mechanisms of ART, Sch B, and CA. Specifically, ART regulates the lncRNA UCA1/miR-184/BCL-2 axis, to inhibit prostate cancer (Zhou S. et al., 2017), while the has-circ-0043256/miR-1252/ITCH axis was involved in the treatment of non-small cell lung cancer by CA (Tian F. et al., 2017). The miR-150/ lncRNA BCYRN1 axis was targeted by Sch B treatment, leading to suppression of cell proliferation in asthma (Zhang X. Y. et al., 2017). All of these complex networks provide foundations for in-depth understanding and broader application of CHMs in the near future.

The interactions between bioactive ingredients of CHMs with ncRNA targets are the subject of intensive and rapidly expanding research. This has helped to reveal the treatment mechanisms underlying the activities of CHMs and offers promising complementary and alternative treatments for diseases, based on scientific research. Although some previous reviews have revealed the increasing importance of bioactive ingredients (Huang et al., 2016; Lelli et al., 2017) or CHMs (Hong et al., 2015) in the treatment of diseases by targeting ncRNA. However, most of them mainly focused on one kind of ncRNA (particularly miRNA), or are limited to a specific disease (mostly cancer) (Mohammadi et al., 2017; Mirzaei et al., 2018); rather than overall and comprehensive ncRNA targets, ceRNA crosstalk and corresponding mechanisms. As a result, we consider it's necessary to make a systematic review about the treatment mechanisms of bioactive ingredients from CHMs by targeting miRNA, lncRNA, and circRNA. From our review, it can be seen that studies are currently in initial and exploratory phases, and several critical problems remain. First, various individual ncRNA molecules are targets of CHM bioactive ingredients; however, recent results are far from sufficient to allow understanding of the complex regulatory interactions between circRNA, lncRNA, miRNA, and mRNA in the treatment of diseases. Second, the metabolism of drugs in single cell lines and animals may differ from that in the human body; therefore, results based on basic research require further verification in clinical trials. Third, each CHM generally contains numerous ingredients and a TCM clinical prescription often consists of several CHMs; therefore, multiple targets and ceRNA crosstalk must occur and the study of classic TCM formulae will further complicate the picture.

In conclusion, ncRNAs are potential targets of CHMs and understanding of ceRNA crosstalk has helped to reveal the complex mechanisms underlying multi-target and multi-level regulation of bioactive ingredients from CHMs. Therefore, CHM ingredients represent new and promising choices for future alternative disease treatments.

## Author Contributions

JW and JL designed the study. YD, HC, and JG conducted searches and extracted the data. YL and YD analyzed the data. YD wrote the manuscript.

### Conflict of Interest Statement

The authors declare that the research was conducted in the absence of any commercial or financial relationships that could be construed as a potential conflict of interest.

## Appendix

**Table:** Chem. 1–43 Chemical formulae of bioactive ingredients from CHMs.

*Chem. 1 Berberine*	*Chem. 2 Artesunate*
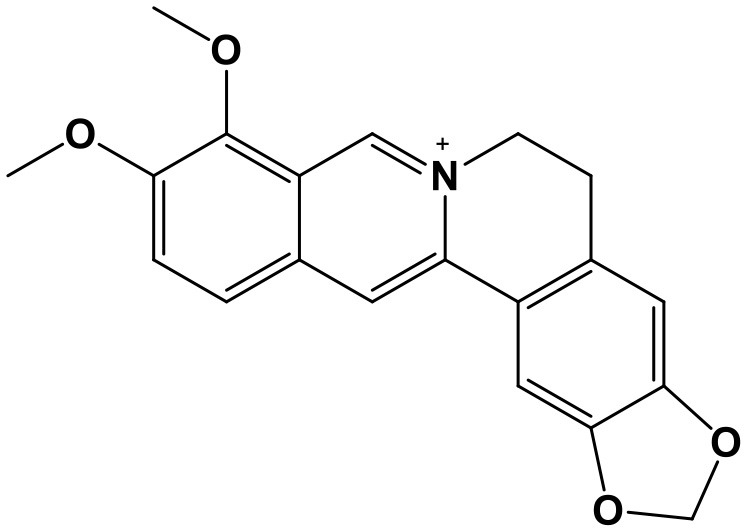
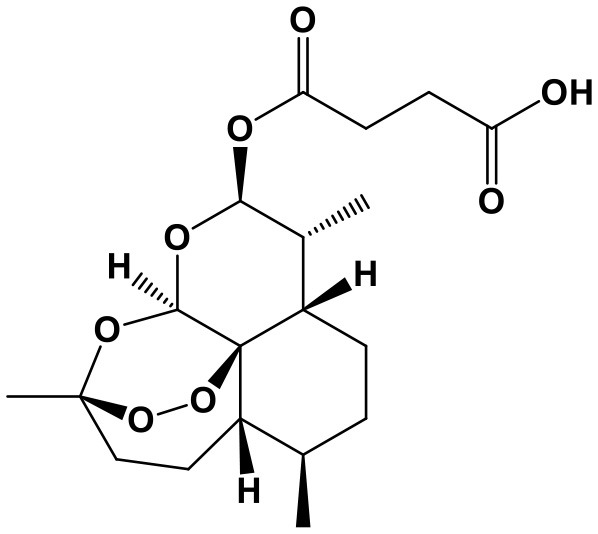
*Chem. 3 Triptolide*	*Chem. 4 Triptonide*
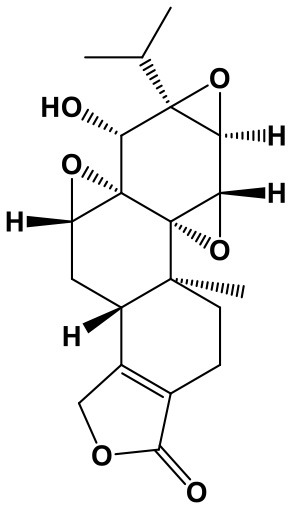
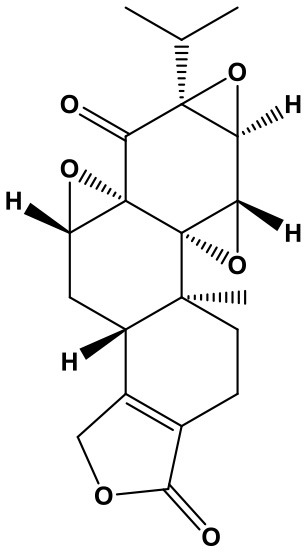
*Chem. 5 Ailanthone*	*Chem. 6 Cordycepin*
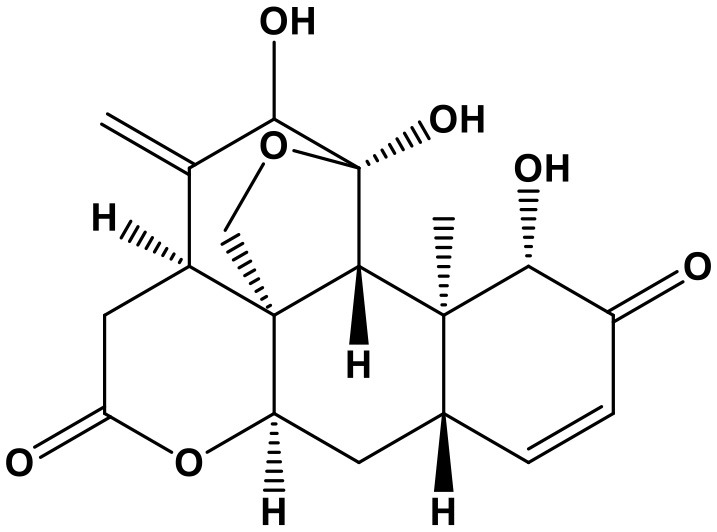
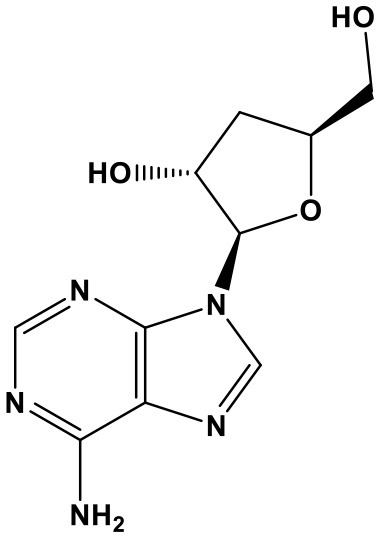
*Chem. 7 Soya-cerebroside*	*Chem. 8 Tubeimoside I*
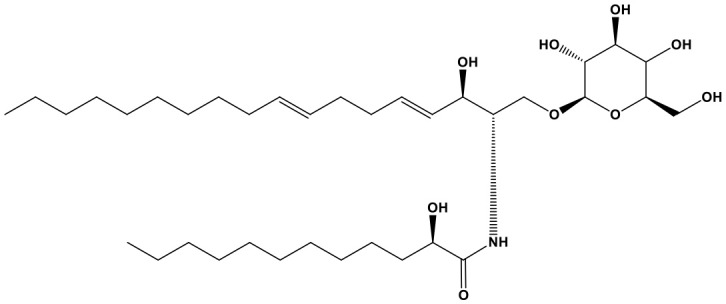
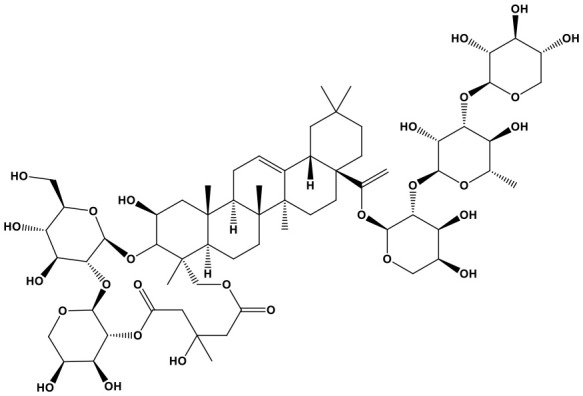
Chem. 9 Oridonin	Chem. 10 Curcumin
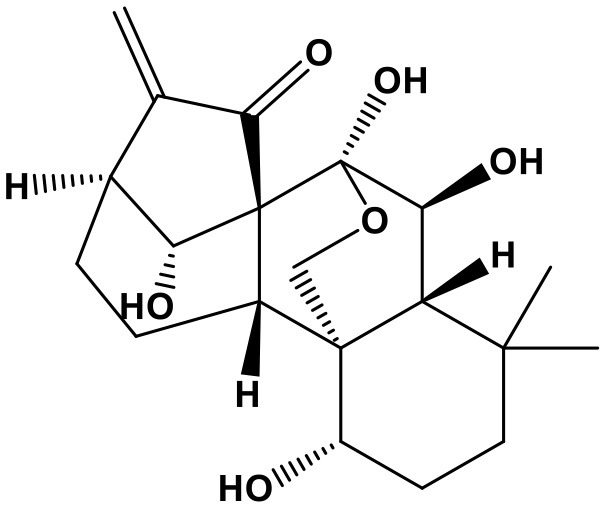
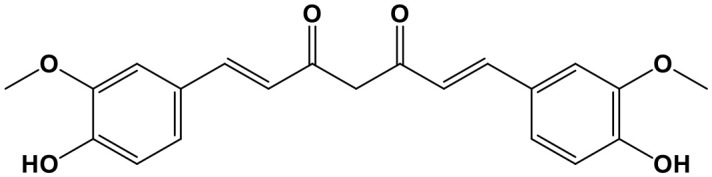
Chem. 11 Shikonin	Chem. 12 Paeoniflorin
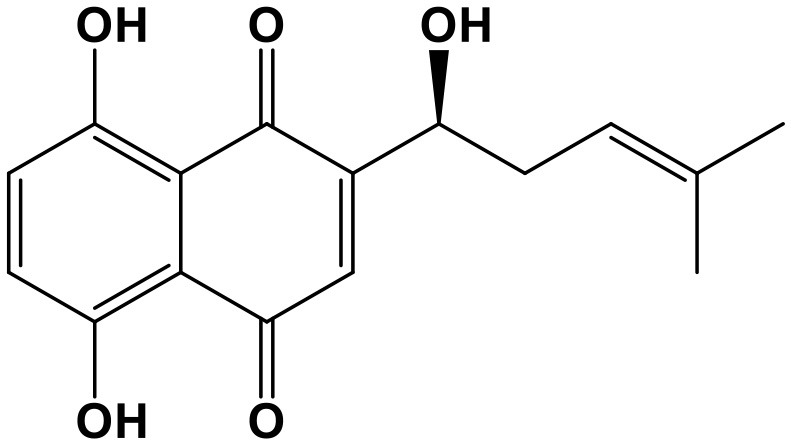
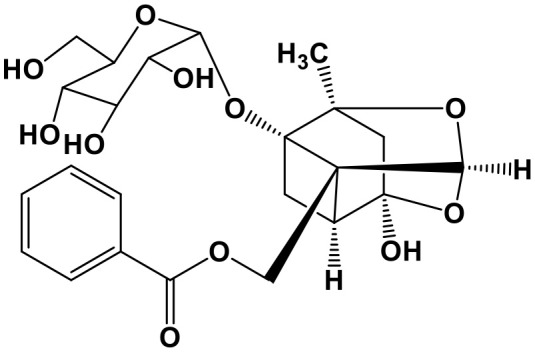
Chem. 13 Honokiol	Chem. 14 Schiscandrin B
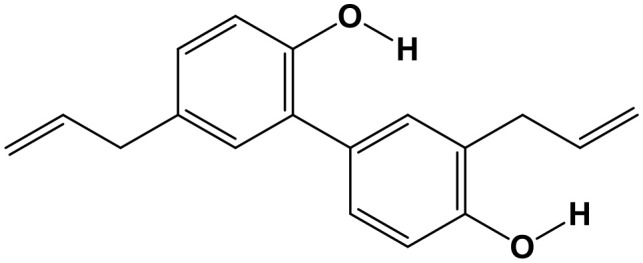
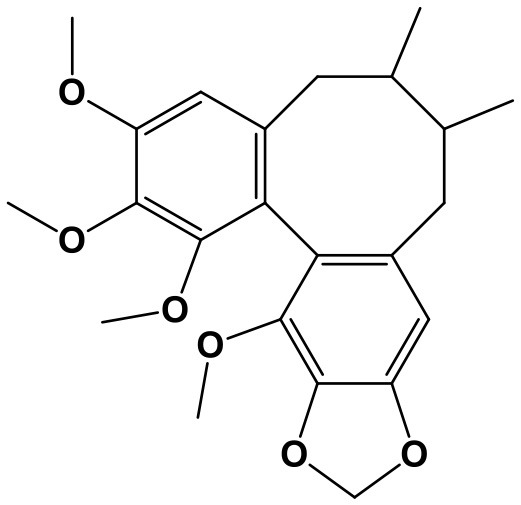
Chem. 15 Resveratrol	Chem. 16 Soybean isoflavones
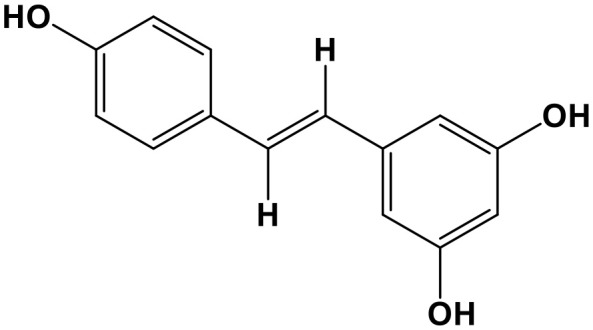
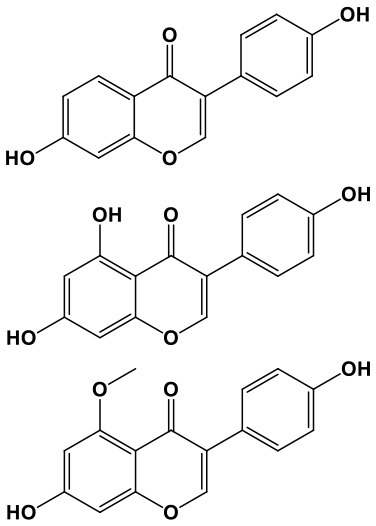
Chem. 17 Matrine	Chem. 18 Corylin
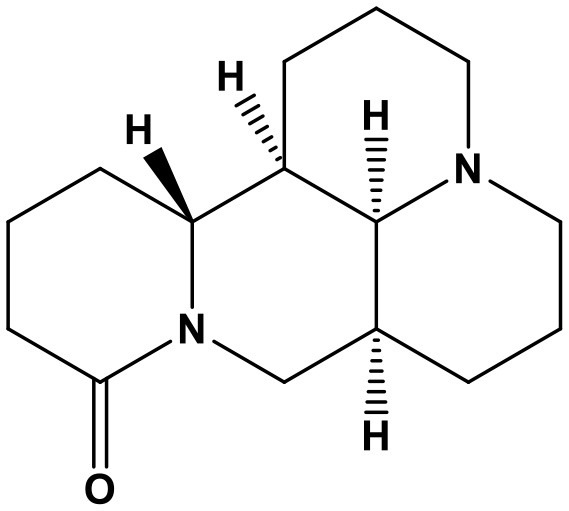
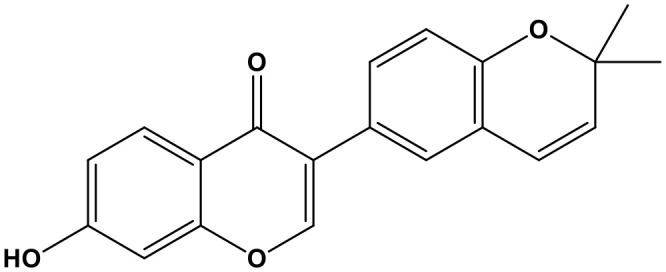
Chem. 19 Tanshinones UA	Chem. 20 Magnesium lithospermate B
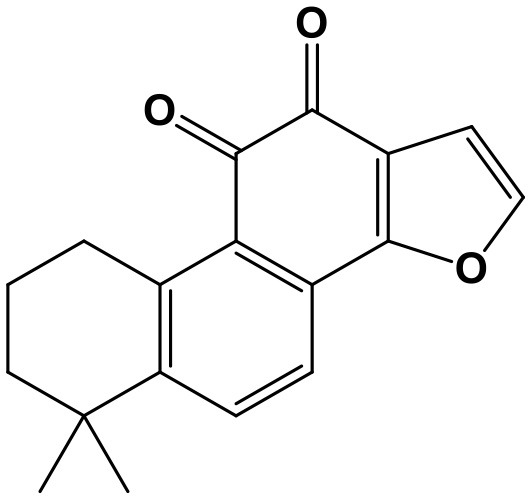
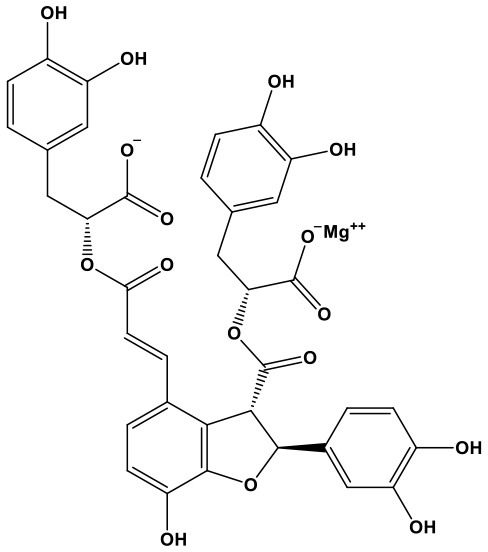
Chem. 21 Baicalin	Chem. 22 Cinnamaldehyde
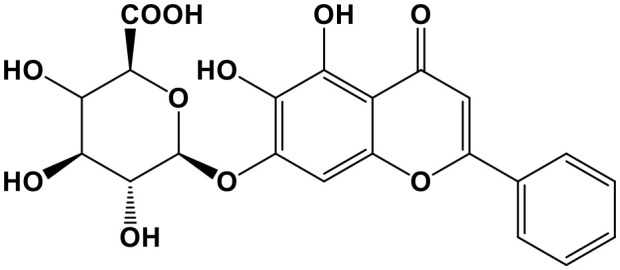
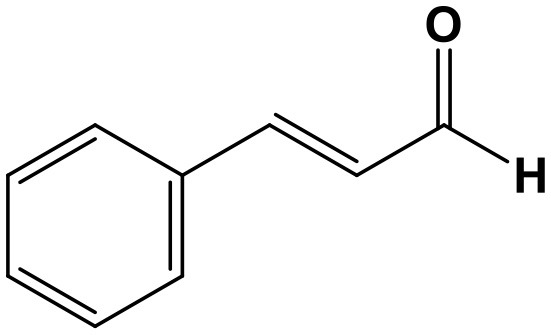
Chem. 23 Geniposide	Chem. 24 Carvacrol
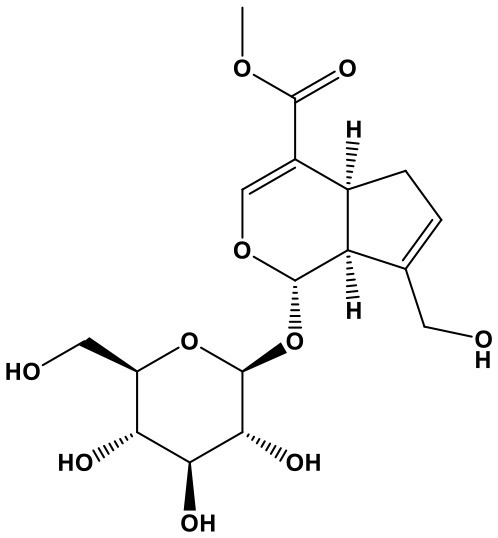
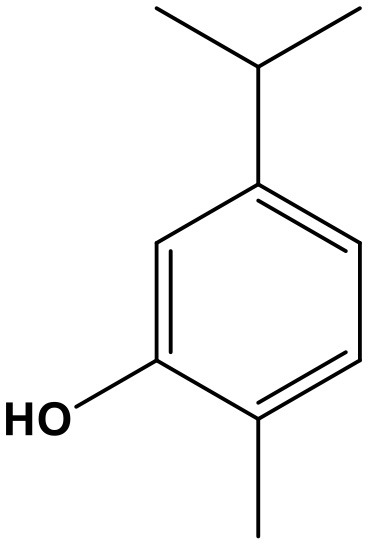
Chem. 25 Thymol	Chem. 26 3-acetyl-11-keto-a-boswellic acid
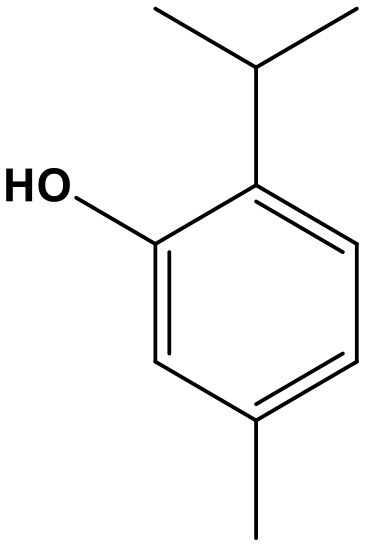
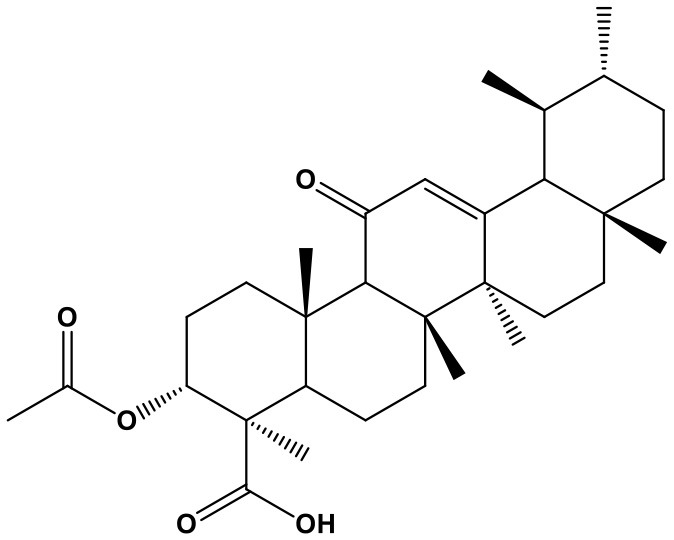
Chem. 27 Sinapic acid	Chem. 28 Polydatin
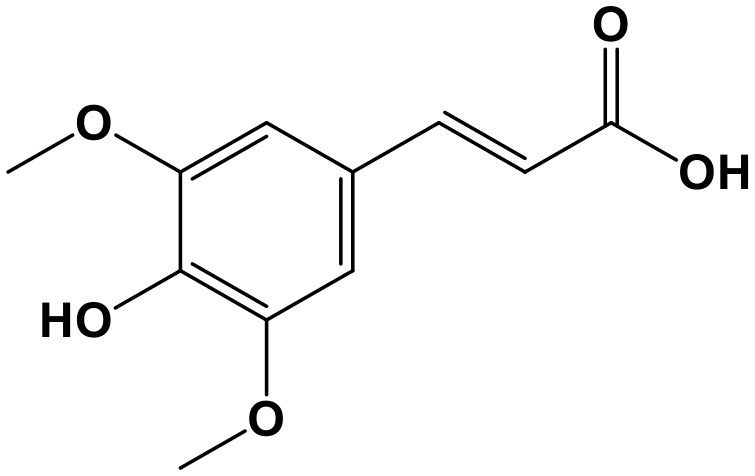
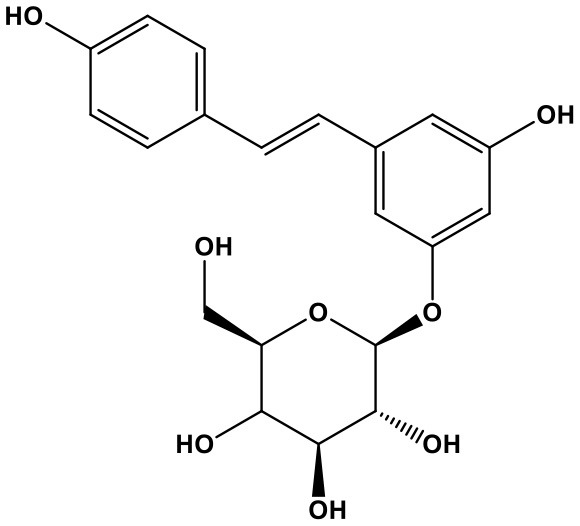
Chem. 29 Ampelopsin	Chem. 30 Icariine
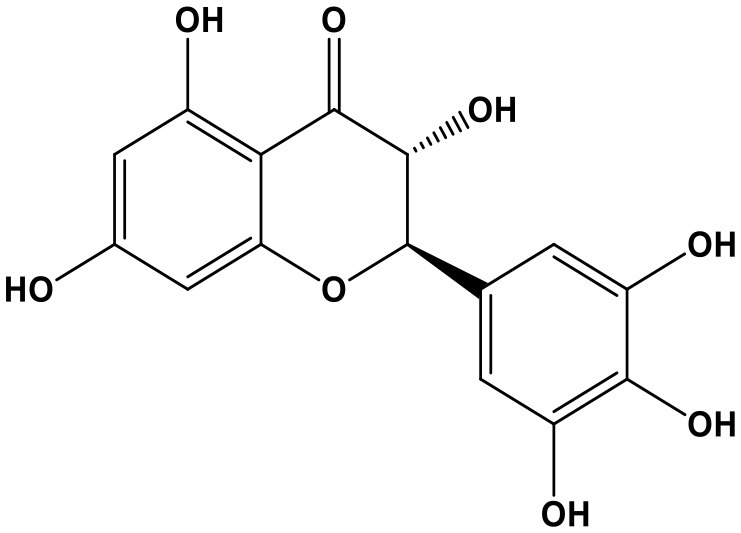
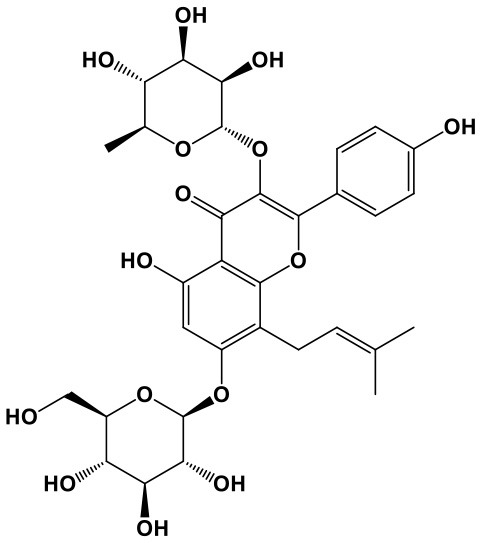
Chem. 31 Ginsenosides	Chem. 32 Salidroside
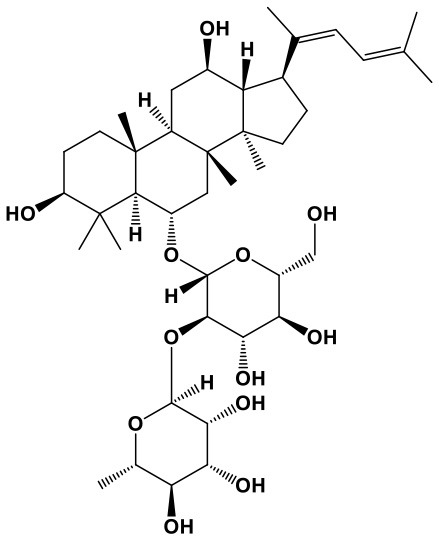
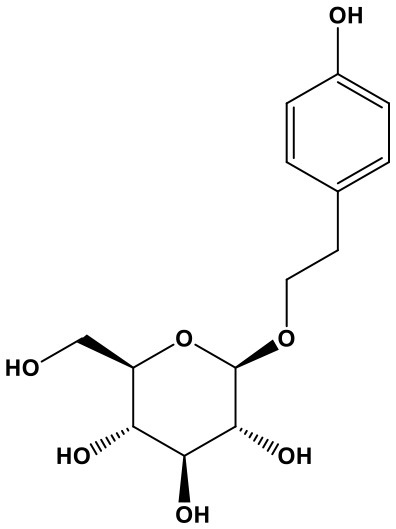
Chem. 33 Phlorizin	Chem. 34 Osthole
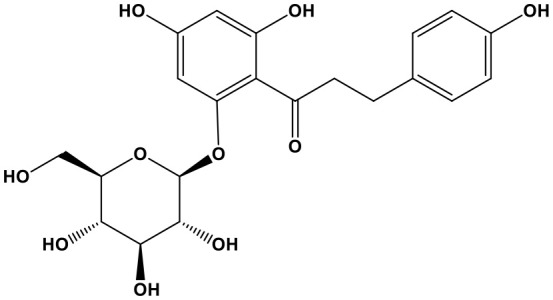
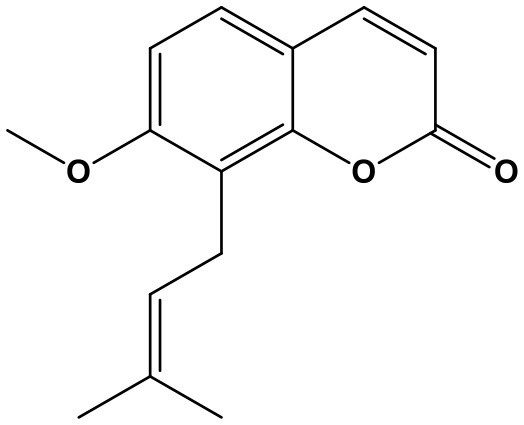
Chem. 35 Panax Notoginseng Saponins	Chem. 36 Tetrandrine
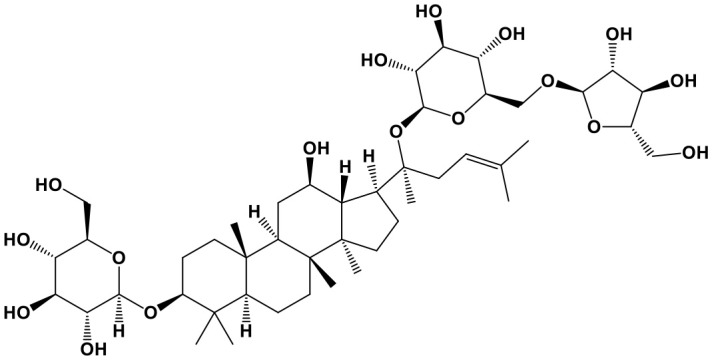
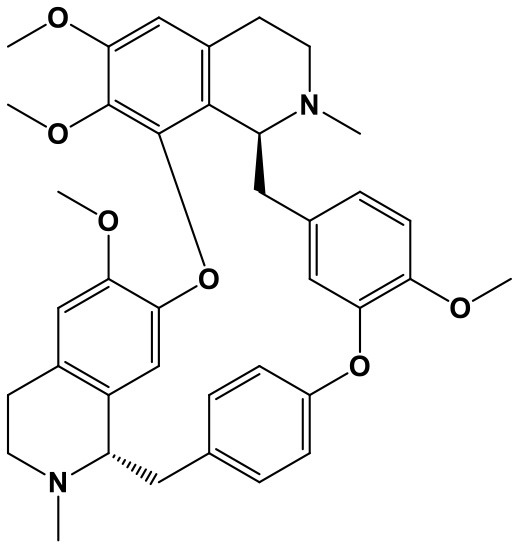
Chem. 37 Leonurine	Chem. 38 Calycosin
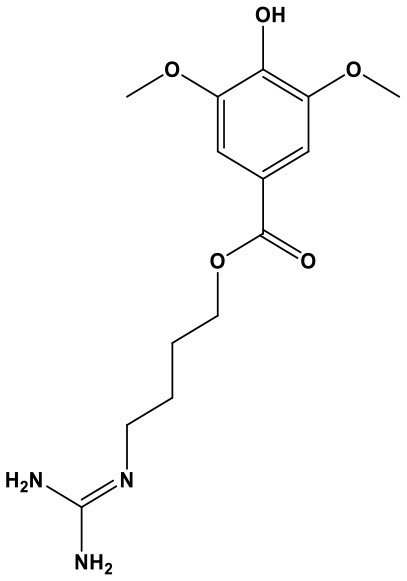
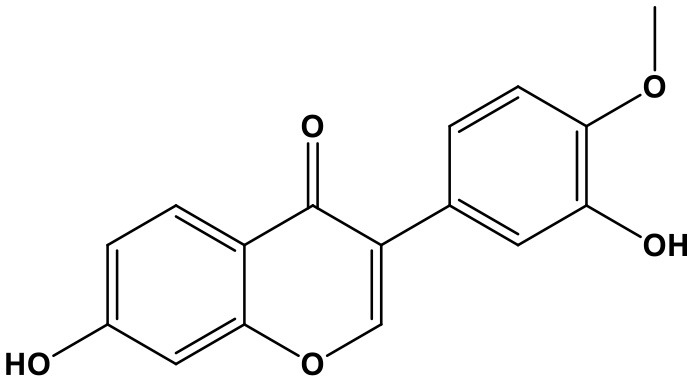
Chem. 39 Astragaloside IV	Chem. 40 Paeonol
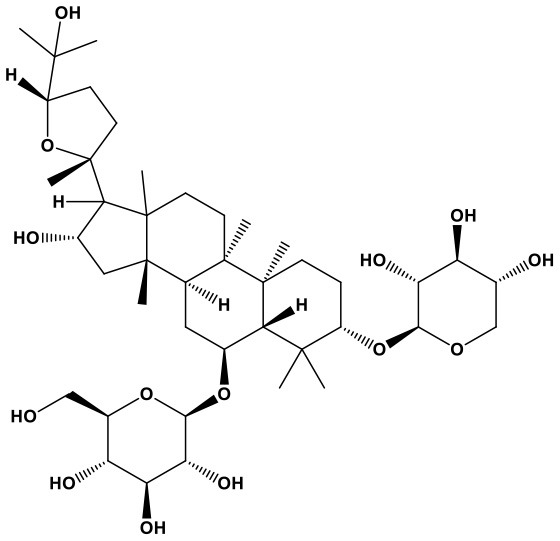
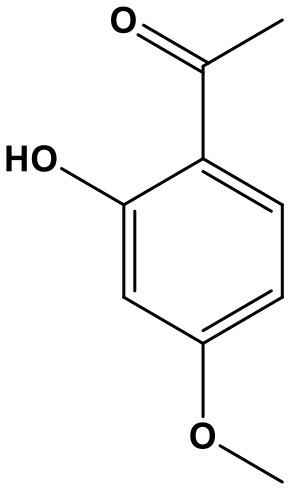
Chem. 41 Salvianolic acid A	Chem. 42 Andrographolide
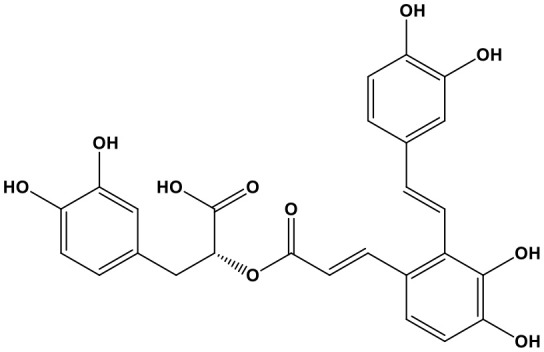
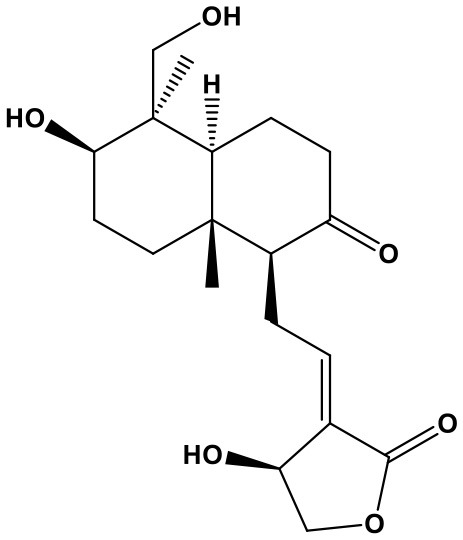
Chem. 43 Puerarin	
